# CAR‐DC combined with CAR‐T therapy for relapsed/refractory acute myeloid leukaemia: Research progress and future perspectives

**DOI:** 10.1002/ctm2.70536

**Published:** 2025-11-25

**Authors:** Rui Zhang, Jinlin Zhang, Hongkai Zhang, Mingfeng Zhao

**Affiliations:** ^1^ Department of Hematology Tianjin First Central Hospital Tianjin China; ^2^ The First Central Clinical College of Tianjin Medical University Tianjin China; ^3^ State Key Laboratory of Medicinal Chemical Biology and College of Life Science Nankai University Tianjin China

**Keywords:** acute myeloid leukaemia, CAR‐DCs, CLL1 CAR‐T

## Abstract

Acute myeloid leukaemia (AML) remains the most common type of leukaemia in adults. Despite advances in conventional therapies, high relapse rates persist, underscoring the need for novel approaches such as chimeric antigen receptor T (CAR‐T) cell therapy. C‐type lectin‐like molecule‐1 (CLL1)‐targeted CAR‐T emerges as a promising treatment for relapsed/refractory (R/R) AML. Although approximately 70% patients achieved remission, only a subset achieved minimal residual disease‐negative remission, which still has much room for improvement. The main reasons for the failure of CLL1 CAR‐T‐cell therapy include: (1) persistence of CLL1‐negative AML cells persist due to antigen escape; (2) downregulation of interleukin (IL)‐12 and other cytokines by the immunosuppressive tumour microenvironment (TME), contributing to the exhaustion of both endogenous T cells and CLL1 CAR‐T cells.

We synthesise a combination of CAR‐engineered dendritic cells (CAR‐DCs) and CLL1 CAR‐T cells to overcome current limitations. CAR‐DCs enhance antigen cross‐presentation to activate endogenous T cells against antigen‐negative clones, secrete immunostimulatory cytokines (e.g., IL‐12) to sustain CAR‐T activity, and remodel the TME. Key challenges involve optimising CAR designs (e.g., incorporating Fms‐like tyrosine kinase 3 ligand [FLT‐3L] or CD40 signalling domains), mitigating toxicity and establishing clinical administration protocols.

In this review, a focused discussion was provided on the specific challenges limiting CLL1‐targeted CAR‐T‐cell therapy in R/R AML, namely antigen escape and the TME, and a novel combination strategy of CAR‐DCs with CLL1 CAR‐T cells was proposed as a promising approach to mitigate these barriers. Here, the rationale, current research advances, and future perspectives of this synergistic strategy were critically examined.

**Highlights:**

- Our earlier clinical trials showed that C‐type lectin‐like molecule‐1 (CLL1)‐targeted therapy for refractory/relapse acute myeloid leukaemia (AML) was validated, which still has a considerable room for improvement.
- We summarise the clinical trials and basic research on the dendritic cell (DC) therapy and chimeric antigen receptor‐engineered DC (CAR‐DC) therapy.
- We explored the synergistic mechanism and prospects of CLL1 CAR‐DC cells combined with CLL1 CAR‐T cells in AML.

## Graphical abstract

1. Chimeric antigen receptor T (CAR‐T) therapy has made some progress in the treatment of acute myeloid leukaemia (AML), among which C‐type lectin‐like molecule‐1 (CLL1) CAR‐T‐cell therapy has shown certain efficacy in the treatment of relapsed/refractory (R/R) AML. However, 30% of patients exhibit primary treatment resistance. Key factors limiting the efficacy of CAR‐T‐cell therapy include antigen escape, interleukin (IL)‐12 and tumour microenvironment (TME).

2. Dendritic cell (DC) mediates antitumour immunity by phagocytosing tumour material, processing tumour antigens and presenting peptide‐MHC complexes to activate tumour specific. CAR‐engineered DCs (CAR‐DCs) can specifically recognise tumour antigens, efficiently phagocytose CAR‐targeted tumour cells and debris, and subsequently activate endogenous tumour‐specific T‐cell responses. Activated CAR‐DC cells can secrete IL‐12 that may help counteract the TME and enhance immune cell function.

3. CAR‐DC cells interaction with CAR‐T cells significantly enhanced anti‐AML cytotoxic activity. CAR‐DC cells phagocytoses AML cells via CAR targeting and secretes IL‐12; activated endogenous T cells and CAR‐T cells work together to eliminate both CLL1‐positive and CLL1‐negative AML clones. Therefore, CAR‐DC and CAR‐T therapy have a good complementarity.

4. The selection and design of the intracellular signal transduction region of CAR is the key in DC cell therapy. The CAR‐DC cells with an FLT3‐integrated intracellular domain enabled conventional DC differentiation and enhanced cross‐presentation of tumour antigens. The CD40 signalling cascade upregulates MHC class II, co‐stimulatory molecules and cytokines.

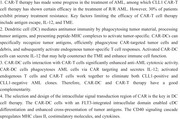

## 1 CURRENT RESEARCH STATUS OF acute myeloid leukaemia TREATMENT

The conventional treatment of acute myeloid leukaemia (AML) mainly includes chemotherapy, targeted therapy and haematopoietic stem cell transplantation, but most patients face the risk of relapse.^1^, ^2^ Chimeric antigen receptor T (CAR‐T) therapy represents a promising novel approach for relapsed/refractory (R/R) AML, although significant challenges remain. Currently, CAR‐T‐cell therapy has achieved impressive clinical outcomes in haematologic malignancies, including acute lymphoblastic leukaemia, lymphoma and multiple myeloma, by targeting CD19, CD22 and b‐cell maturation antigen (BCMA).^3^, ^4^, ^5^, ^6^ However, CAR‐T‐cell therapy remains clinically immature for AML, with limited overall efficacy in current studies.^7^, ^8^ The primary reasons include the lack of specific targets,^9^, ^10^ the immunosuppressive tumour microenvironment (TME)^11^, ^12^ and antigen escape.^13^, ^14^, ^15^ Early CAR‐T therapies for AML targeted CD33 and CD123, but their toxicity or poor efficacy has limited their clinical application.^7^, ^16^, ^17^ Other potential targets include C‐type lectin‐like molecule‐1 (CLL1), FLT3, NKG2D, CD7, CD38, etc.^18^, ^19^, ^20^, ^21^, ^22^, ^23^ CLL1‐targeted CAR‐T cells have demonstrated promising efficacy in the treatment of R/R AML.

## 2 THERAPEUTIC EFFICACY OF CLL1 CAR‐T CELLS IN RELAPSED/REFRACTORY AML

CLL1 is a C‐type lectin‐like receptor that is highly expressed on leukaemia stem cells (LSCs) (about 45%) and leukaemia progenitor cells (77.5%‒92%). Tashiro et al. developed the first CLL1 CAR‐T cells, which selectively killing leukemic progenitor cells and leukaemia cells.^24^ CLL1 CAR‐T cells have shown superior AML killing in vitro and in mice.^18^ A case report described complete remission (CR) in a 10‐year‐old patient after CLL1 CAR‐T therapy.^25^ Our group reported a 70% CR rate in 10 adult AML patients treated with CLL1 CAR‐T cells.^26^ At present, a total of 48 patients received CLL1 CAR‐T‐cell therapy. All patients underwent efficacy evaluation between days 14 and 16 post‐infusion. CR was achieved in 34 patients (70.83%), including 13 with minimal residual disease (MRD)‐positive CR and 21 with MRD‐negative CR. The remaining 14 patients (29.17%) showed no response to the treatment. Regarding adverse events, cytokine release syndrome (CRS) and immune effector cell‐associated neurotoxicity syndrome (ICANS) occurred in 95.83% and 20.83% of patients, respectively. Severe (grade 3/4) CRS and ICANS were observed in 41.67% and 10.42% of patients. Haematologic toxicities were the most common complications, with near‐universal incidence of leukopaenia, granulocytopaenia, anaemia and thrombocytopaenia. Notably, severe and prolonged granulocytopaenia significantly increased the risk of infectious complications. Among the reported infections, 38 patients experienced bacterial infections, while viral and fungal infections were documented in nine and 10 patients, respectively. Comparative analysis further revealed that patients with severe CRS (grade 3/4) exhibited more pronounced cytokine release, particularly elevated levels of interleukin (IL)‐2, IL‐6, IL‐10, C‐reactive protein and ferritin, compared to those with mild CRS (grade 1/2).^27^ Although CLL1‐targeted CAR‐T cells have shown clinical efficacy in R/R AML, approximately 30% of patients exhibit primary treatment resistance. Furthermore, only 50% of responders achieve MRD‐negative remission, underscoring the need for enhanced therapeutic strategies.

The key limitations include the following: (1) antigen escape—AML cells with low CLL1 expression are difficult for CAR‐T cells to recognise, and relapsed patients frequently present weak expression of target antigens.^28^, ^29^ (2) TME and IL‐12—the TME reduces the efficacy of CAR‐T cells via myeloid‐derived suppressor cells (MDSCs), regulatory T cells (Tregs) and tumour‐associated macrophages (TAMs).^30^, ^31^, ^32^ Dysregulated cytokines, including IL‐12, further impair CAR‐T‐cell function. Notably, mKRAS‐specific NeoCARs with inducible IL‐12 secretion and T‐cell receptor (TCR) knockout (KO) exhibit strong in vivo antitumour activity and favourable safety.^33^ Co‐expression of IL‐12 and interferon‐gamma (IFN)α2 with CAR enhances the proinflammatory microenvironment and mitigates T‐cell exhaustion.^34^ Moreover, mbIL‐12‐engineered CAR‐T cells have demonstrated safety and efficacy in overcoming the TME.^35^ IL‐12 not only increases CAR‐T cytotoxicity, but also remodels the TME by increasing proinflammatory CD4^+^ T‐cell infiltration, decreasing Tregs, and activating myeloid cells, with minimal systemic toxicity in glioblastoma multiforme‐targeted CAR‐T‐cell therapy.^36^ Therefore, integrated strategies combining CAR‐T modification and TME remodelling are essential for improving clinical outcomes of CLL1 CAR‐T‐cell therapy.

## 3 RESEARCH PROGRESS AND CHALLENGES ASSOCIATED WITH DENDRITIC CELL AND CAR‐ENGINEERED DENDRITIC CELL THERAPY

Dendritic cells (DCs) regulate antitumour immunity by phagocytosing tumour material, processing tumour antigens and presenting peptide‒major histocompatibility complex (MHC) complexes to activate tumour‐specific T cells.^37^, ^38^ Mature DCs directly engage T cells through co‐stimulatory molecules (CD80/CD86), secrete IL‐12 and initiate endogenous tumour‐specific T‐cell priming.^39^, ^40^, ^41^ Mature DCs trigger tumour‐specific CD8^+^ T‐cell immunity by migrating from tumours to lymph nodes, capturing antigens and activating naïve T cells.^42^

Conventional DCs (cDCs) are classified into two major subsets: type 1 (cDC1) and type 2 (cDC2).^43^ cDC1s prime CD8^+^ T cells in draining lymph nodes (dLNs) and activate Toll‐like receptors to induce IL‐12p70 and IFN‐α, promoting Th1‐type immunity. Their presence correlates with favourable prognosis in cancer patients.^44^ cDC2s, more abundant than cDC1s, express CD11c, CSF‐1R, MHC‐II, CD11b, BDCA1 and SIRPα produce various cytokines, such as IL‐23 and IL‐10, and present antigens to CD4^+^ helper T cells, activating Th2 and Th17 subsets.^45^ Novel DC subsets, including LAMP3^+^ DCs^46^, ^47^ and AXL^+^SIGLEC6^+^ (AS) DCs^48^ have been identified. Studies have described DC precursors in bone marrow and blood, showing both shared and distinct regulatory networks, and suggesting underlying heterogeneity within pre‐DCs reflecting early lineage bias.^49^, ^50^

Single‐cell and bulk RNA‐seq revealed enrichment of immunosuppressive CCL22⁺DCs, termed ‘exhausted DCs’, which were reversed by the herbal formula SBJDD and its component berberine; TMEM131‐mediated tumor necrosis factor (TNF) signalling, promoted CCL22⁺DC development, inhibited by berberine.^51^ Lineage tracing showed DC3s arise from monocyte‐DC progenitors via Lyz2⁺Ly6C⁺CD11c^−^ precursors, distinct from DC2 lineage, indicating DC3 as a separate lineage phenotypically related, but developmentally independent from monocytes.^52^ scRNA‐seq of sepsis revealed major immune cell changes and a distinct cDC subcluster with maturation, migration and immunoregulatory signatures, consistent with mregDCs; this sepsis‐induced mregDC subset was validated and shown to activate naïve CD4⁺ T cells while promoting Tregs differentiation.^53^

Functional status depends on maturation: mature DCs express high MHC and co‐stimulatory molecules (e.g., CD80/86), and promote effector T‐cell activation, whereas immature DCs often induce Tregs and immune tolerance. Tumours impair DC maturation to evade immunity. In AML, DC frequency and function are frequently impaired, contributing to disease progression and therapeutic resistance.^54^ cDC1s specifically perform antigen cross‐presentation in vivo and are essential for adaptive antitumour immunity; without cDC1, tumours escape immune elimination.^55^ Due to their superior T‐cell activation and cross‐presentation capacity, cDC1 is a preferred subset for CAR‐DC‐based immunotherapy.

DC/tumour fusion vaccines or tumour lysate‐loaded DCs can activate and sustain antitumour T‐cell responses and expand tumour‐specific T‐cell clones.^56^ A phase II clinical trial (NCT03059485) showed DC/AML fusion vaccination without maintenance led to 73% 2‐year overall survival and 36% progression‐free survival in elderly AML patients.^57^ Eps8‐DCs enhance CD19 CAR‐T function, increasing cytokines, CD107a degranulation and cytotoxicity.^58^ However, DC dysfunction contributes to immune evasion and limits DC vaccine efficacy in elderly patients.^59^ Clinical moDC vaccine efficacy remains variable, limited by antigen‐loading challenges, incomplete maturation and tumour heterogeneity. IL‐12 secretion directly influences endogenous DC1 function.^57^, ^60^, ^61^ cDC vaccines face significant limitations, including the dysfunction of DCs (especially in the elderly), poor and variable efficacy due to challenges in antigen loading and incomplete maturation, and susceptibility to tumour heterogeneity. These limitations underscore the need for advanced strategies such as CAR‐DC platforms. Supporting Information

TME impairs DC function via multiple inhibitory mechanisms, including: (1) induction of DC apoptosis;^62^, ^63^ (2) inhibition of DC maturation and antigen presentation;^64^, ^65^ (3) promotion of tolerogenic DC phenotypes;^66^, ^67^ and (4) downregulation of DC‐recruiting chemokines to limit tumour infiltration.^68^, ^69^, ^70^, ^71^

Given the critical role of functional DCs and their vulnerability in AML, strategies to reprogram or ‘rejuvenate’ DCs have become a key research focus. The DC growth factor Fms‐like tyrosine kinase 3 ligand (Flt3L) enhances T‐cell‐mediated antitumour immunity by expanding and activating DC populations.^55^ Highly activated DCs can induce CD4^+^ T cells to acquire cytotoxic and antitumour functions in aged mice.^58^ PU.1‐, BATF3‐ and IRF8‐mediated DC reprogramming reduces exhaustion and increases memory‐ and stem‐cell‐like T‐cell infiltration.^72^ KO of BCL9/BCL9L in cDC1 enhances CD8^+^ T‐cell activation, antigen presentation, epitope expansion and promotes antitumour activity.^73^, ^74^ Although promising approaches such as Flt3L expansion and transcriptional reprogramming via PU.1, BATF3 and IRF8 have been developed, DC reprogramming still faces considerable challenges. Key limitations include the translational gap between mouse models and human patients, the difficulty and safety concerns of controlling genetic or transcriptional programs in clinical settings, and the instability of the reprogrammed states in the immunosuppressive TME.

Moreover, CAR‐DCs can specifically recognise tumour antigens, efficiently phagocytose CAR‐targeted tumour cells and debris, and subsequently activate endogenous tumour‐specific T‐cell responses. Antitumour T‐cell responses can directly eliminate CAR‐targeted antigen‐positive tumours and indirectly eliminate antigen‐negative tumours (which are not directly recognised by the CAR) through cross‐presentation and epitope spreading.^69^, ^75^ Activated CAR‐DCs secrete immunostimulatory cytokines such as IL‐12.^35^, ^69^, ^76^, ^77^ The intracellular signalling domain of the CAR can remain continuously activated during tumour recognition, enabling DCs to sustain homeostasis and resist the suppressive microenvironment, allowing tolerant DCs to reacclimate.

The mechanism by which CAR‐DCs overcome antigen escape and target CAR antigen‐negative cells is based on their unique capacity for antigen cross‐presentation and subsequent epitope spreading. (1) Phagocytosis of antigen‐positive cells: CAR‐DCs use their CAR to specifically recognise and phagocytose CAR‐positive (e.g., CLL1⁺) AML cells. (2) Cross‐presentation and endogenous T‐cell activation: after phagocytosis, CAR‐DCs process whole‐cell contents and present a broad repertoire of tumour‐derived peptides via MHC‐I to activate endogenous, CD8⁺ T cells. (3) Elimination of antigen‐negative clones via epitope spreading: these activated endogenous T cells are polyclonal and can recognise multiple tumour antigens, allowing them to eradicate AML cell populations that escape CAR‐T therapy by losing the target antigen (e.g., CLL1‐negative cells). This immune expansion from a single antigen to additional antigens is termed epitope spreading.

The process by which CAR‐DCs target and eliminate CAR antigen‐negative cells is as follows: CAR‐mediated phagocytosis → broad antigen processing and cross‐presentation → activation of endogenous T cells against multiple tumour antigens → elimination of antigen‐negative clones via epitope spreading.

## 4 VALIDATION OF TARGET SPECIFICITY USING GENE KO MODELS

In vitro validation often involves the use of CRISPR‐Cas9 or RNA interference to KO the target antigen (e.g., CLL1) in AML cell lines. For instance, CLL1‐KO AML cells show significantly reduced susceptibility to CLL1‐targeted CAR‐T‐cell killing, while retaining sensitivity to CAR‐T cells targeting alternative antigens (e.g., CD33 or CD123).^18^, ^24^ This confirms the antigen specificity of the CAR construct. Similarly, when CAR‐DCs are co‐cultured with CLL1‐KO AML cells, their phagocytic capacity and subsequent T‐cell activation are markedly diminished, underscoring the dependence on CAR‐mediated recognition.^69^, ^75^

In vivo studies further validate these findings using xenograft models established with CLL1‐deficient AML cells. In such models, CLL1 CAR‐T cells exhibit reduced leukemic control compared to their activity against CLL1‐positive tumours, highlighting the role of antigen expression in therapeutic efficacy.^18^, ^26^ Moreover, in dual‐flank tumour models—where one tumour expresses CLL1 and the other is CLL1‐KO—adoptive transfer of CLL1 CAR‐T cells combined with CAR‐DCs results in preferential regression of the CLL1‐positive tumour, with limited impact on the KO tumour unless epitope spreading occurs via CAR‐DC‐mediated cross‐presentation.^75^, ^82^ These KO models not only confirm the on‐target mechanism of action but also help elucidate escape mechanisms. For example, residual disease in CLL1‐KO models often emerges from antigen‐low or antigen‐negative clones, reinforcing the need for combination strategies that address heterogeneity, such as the incorporation of CAR‐DCs to broaden antigen recognition.

Genetic KO models provide indispensable evidence for the specificity and mechanism of CAR‐DCs and CAR‐T therapies. They validate that targeting CLL1 is both necessary and sufficient for initiating antitumour responses, while also revealing limitations that inform the design of next‐generation combinatorial immunotherapies.

## 5 CAR‐DC COMBINED WITH CAR‐T‐CELL THERAPY

CAR‐DC therapy demonstrates antitumour efficacy in AML models and is under clinical evaluation for solid epithelial malignancies (NCT05631899 and NCT05631886). NCT05631899 is a pilot clinical trial assessing the safety, immune activity and efficacy of an EphA2‐targeting CAR‐DC vaccine loaded with a KRAS mutant peptide (KRAS‐EphA2‐CAR‐DC) plus immune checkpoint inhibitors (ICIs) in patients with locally advanced or metastatic solid tumours. Preclinical findings indicate that engineered CAR‐DCs enhance the cytotoxicity of co‐administered CAR‐T cells in solid tumour mouse models. NCT05631886 is a parallel pilot trial evaluating an EphA2‐directed CAR‐DC vaccine loaded with a TP53 mutant peptide (TP53‐EphA2‐CAR‐DCs) plus ICIs in solid tumours or R/R lymphomas.

Multiple studies indicate that intratumoural DC delivery can safely augment CAR‐T‐cell activity and alleviate the immunosuppressive TME. In vitro differentiation of DCs expressing 4‐1BB is enriched for the CD141^+^/ClEC9A^+^ DC subset. CAR‐DC and CAR‐T‐cell interactions synergistically enhance anti‐AML cytotoxicity.^78^ DC‐derived cytokines, such as IL‐12 and type I IFNs, provide additional T‐cell stimulation during antigen presentation, while CAR‐T cells retain their intrinsic cytotoxic capacity independent of DCs.^79^ These data indicate that intratumoural CAR‐DC delivery establishes an ‘immunological niche’. CAR‐DCs secrete IL‐12 and type I IFNs to reverse T‐cell exhaustion and remodel the TME. This synergy addresses CAR‐T‐cell limitations, including inadequate tumour infiltration and antigen loss, through DC‐mediated antigen spreading and localised immune activation.

Studies further demonstrate that 4‐1BB‐engineered autologous DCs enhance the efficacy of anti‐CD33 CAR‐T cells in AML by secreting cytokines and facilitating CAR‐T‐cell recruitment to the bone marrow niche.^80^ The cooperative interaction between CAR‐DCs and CAR‐T cells may represent a powerful strategy to improve antitumour immunotherapy.

**FIGURE 1:**
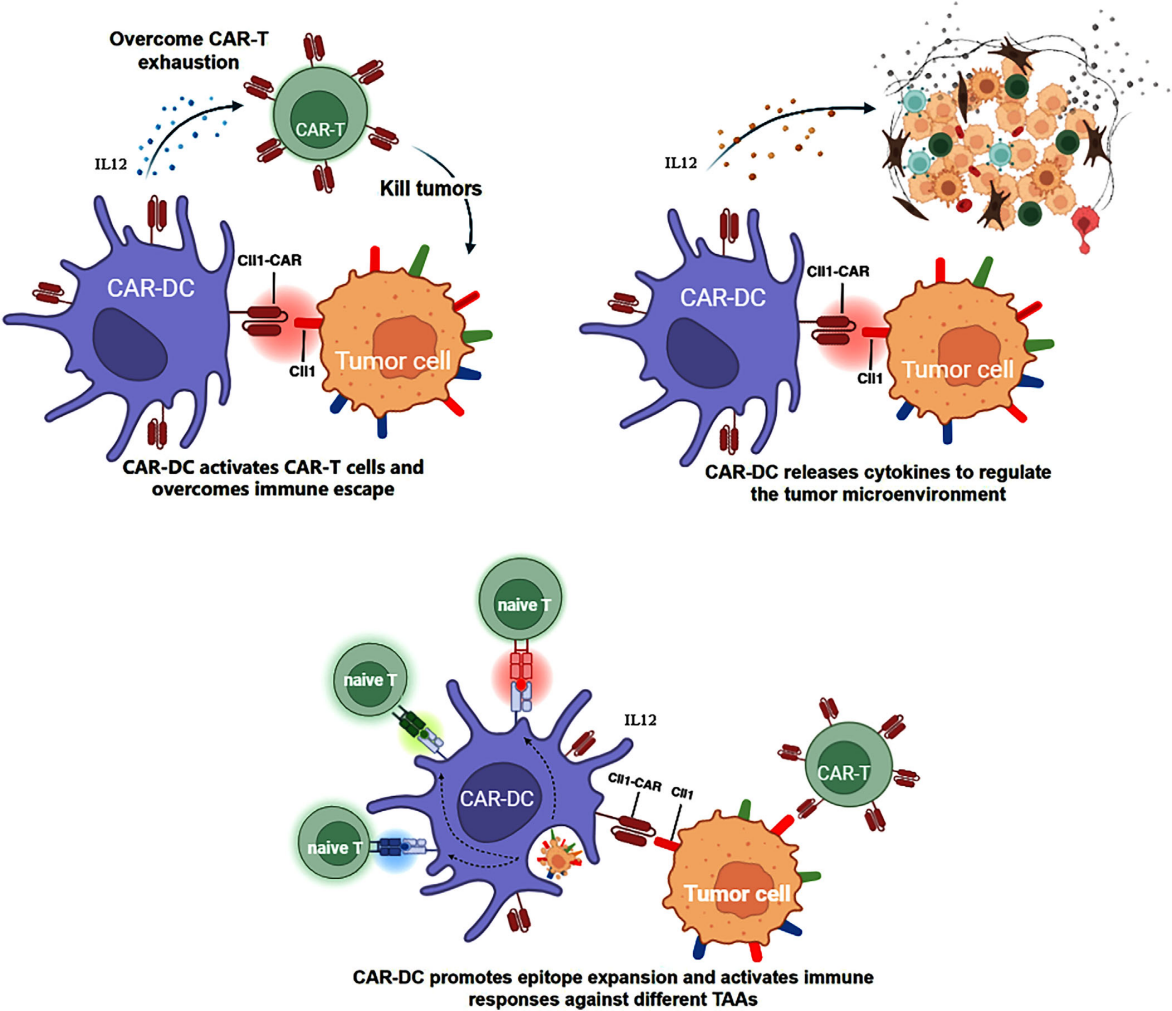
Mechanistic overview of chimeric antigen receptor‐engineered dendritic cell (CAR‐DC) cells and chimeric antigen receptor T (CAR‐T) cell collaboration: (1) CAR‐DC cells phagocytoses acute myeloid leukaemia (AML) cells via CAR targeting, processes antigens and cross‐presents tumour‐derived peptides via major histocompatibility complex (MHC)‐I/II to activate endogenous T cells. (2) CAR‐DC secretes cytokines (e.g., interleukin [IL]‐12) to support the activation, proliferation and persistence of C‐type lectin‐like molecule‐1 (CLL1) CAR‐T cells, potentially reducing exhaustion and promoting a memory phenotype. (3) Activated endogenous T cells (primed by CAR‐DC) and CAR‐T cells work together to eliminate both CLL1‐positive and CLL1‐negative (via epitope spreading) AML clones.

Mechanistic overview of CAR‐DC and CAR‐T‐cell collaboration (Figure 1): (1) CAR‐DCs phagocytose AML cells via CAR targeting, process antigens and cross‐present tumour‐derived peptides via MHC‐I/II to activate endogenous T cells.^40^, ^41^ (2) CAR‐DCs secrete cytokines such as IL‐12 to promote CAR‐T‐cell support the activation, proliferation and persistence, reducing exhaustion and supporting memory formation.^35^, ^40^, ^79^, ^81^ (3) Activated endogenous T cells and CAR‐T cells cooperatively eradicate both CLL1‐positive and CLL1‐negative AML populations through direct cytotoxicity and epitope spreading.^82^, ^83^ Comparative characteristics of CAR‐DCs are summarised in Table 1.

**TABLE 1:** Comparative characteristics of chimeric antigen receptor‐engineered dendritic cells (CAR‐DCs).

Target antigen	CAR‐DC structure	Delivery methods	Animal models used	Cytokine domains	Antitumour outcomes
CD33^71^	DC precursors were transduced with a CAR (pCCL‐anti‐CD33‐4‐1BB‐CD3ζ‐T2A‐GFP). The differentiation of DC in vitro employed Flt3L/GM‐CSF/IL‐4	Lentivirus transduction	NSG AML mice model	Differentiation of DC in vitro employed Flt3L/GM‐CSF/IL‐4	CAR‐DC and CAR‐T cells enhances cytotoxic cytokine production in response to DC‐derived IL‐12. These combined effects resulted in improved anti‐CD33 CAR‐T cytotoxicity in vitro and in vivo NSG AML mice model.
KRAS‐EphA‐2 [clinical trail: NCT05631899]	KRAS‐EphA‐2‐CAR‐DC contain a scFv domain targeting EphA2 antigen and KRAS mutant peptide, CD8a transmembrane, tandem DC‐specific activation domains	Vaccine	Clinical trial for solid tumours	IL‐2, IL‐6, IL‐8, IL‐10, IL‐12 (p70), TNF‐α	KRAS‐EphA‐2‐CAR‐DC can suppress the growth of tumours expressing the correlated KRAS mutant in animal models. In addition, the combination of the immune checkpoint inhibitors could further reverse immunosuppressive TME and globally activate T‐cell responses.
P53‐EphA‐2 [clinical trail: NCT05631886]	P53‐EphA‐2‐CAR‐DC contain a scFv domain targeting EphA2 antigen, CD8a transmembrane, tandem DC‐specific activation domains	Vaccine	Clinical trial for R/R lymphomas	IL‐2, IL‐6, IL‐8, IL‐10, IL‐12 (p70), TNF‐α	P53 (R273H, R175H, R248Q or R249S)‐EphA‐2‐CAR‐DC can suppress the growth of tumours expressing the correlated TP53 mutant in animal models. In addition, the combination of the immune checkpoint inhibitors could further reverse immunosuppressive TME and globally activate T‐cell responses.
BCMA^74^	BCMA‐CAR‐DC: CD8a signal peptide, BCMA VHH antibodies, CD8a transmembrane, CD40 intracellular domain	Lentiviral transduction	Multiple myeloma	IL‐12, TNF‐α	CAR‐DC cells specifically target multiple myeloma cells overexpressing BCMA receptors. They selectively bind to these malignant cells and enhance TRAIL‐mediated apoptosis, thereby effectively eliminating multiple myeloma cells for therapeutic purposes.
CD123^75^ CD33	scFv + CD8a transmembrane + intracellular domain (CD3ζ; 4‐1BB + CD3ζ; FLT3L + CD3ζ)	Lentiviral transduction	R/R AML	IL‐12, TNF‐α	Selecting the appropriate intracellular domain (e.g., 4‐1BB, FLT3 or CD40) of CAR can induce the mature phenotype of CAR‐DC when recognising the tumour, and overcome the acclimation of tolerant DC cells by the suppressive TME.

Abbreviations: AML, acute myeloid leukaemia; Flt3L, Fms‐like tyrosine kinase 3 ligand; IL, interleukin; R/R, relapsed/refractory; TME, tumour microenvironment; TNF, tumor necrosis factor.

Therefore, CAR‐DCs and CAR‐T‐cell therapy exhibit strong complementarity, and can enhance CAR‐T‐cell function while overcoming immune escape to reduce tumour recurrence. The limitations of the CAR‐DC and CAR‐T‐cell combination strategy can be summarised as follows: (1) early‐stage clinical evidence—the efficacy of this combination is primarily supported by preclinical data. Human clinical evidence remains limited to early‐phase pilot studies (e.g., NCT05631899 and NCT05631886), and definitive therapeutic value has not yet been established. (2) Logistical and manufacturing complexity—this therapeutic modality requires the production, quality control and administration of two advanced cellular products (CAR‐DCs and CAR‐T cells), substantially increasing complexity, cost and barriers to scalable manufacturing compared with single‐cell products. (3) Unverified synergy in human tumours—although animal models support the creation of an ‘immunological niche’ capable of reversing T‐cell exhaustion and overcoming the immunosuppressive TME, consistent confirmation in human tumours is still required. (4) Safety uncertainties—dual activation of engineered immune cell populations may amplify toxicity risks, including exacerbated CRS. The full safety profile of this combined approach remains insufficiently characterised. Table 2

**TABLE 2:** Novel cellular immunotherapies: mechanisms and clinical status.

Therapeutic modality	Mechanism of action	Target antigen(s)	Efficacy/response (clinical/preclinical)	Key advantages	Key limitations	Clinical status
CLL1 CAR‐T	CAR‐T cells directly kill CLL1^+^ AML cells	CLL1	∼70% CR (34/48 pts); ∼44% MRD‐ CR (21/48 pts)^26^, ^27^	Targets LSCs; clinically validated	Antigen escape; TME suppression; high CRS/ICANS rates	Phase I/II
CAR‐DC + CAR‐T	CAR‐DCs phagocytose tumour, cross‐present antigens, secrete cytokines; synergise with CAR‐T	CLL1 (CAR‐DC + CAR‐T)	Preclinical: enhanced cytotoxicity and epitope spreading^78^, ^80^	Overcomes antigen escape; remodels TME; activates endogenous T cells	Logistical complexity; unproven clinical synergy; potential CRS amplification	Early‐phase trials (e.g., NCT05631899)
DC/AML fusion vaccine	DCs loaded with AML antigens activate endogenous T cells	Multiple tumour‐associated	73% 2‐year OS in elderly AML (phase II)^57^	Broad antigen response; favourable safety	Limited efficacy in elderly; variable response; DC dysfunction	Phase II
Dual‐target CAR‐T (e.g., CD33 + CD123)	CAR‐T cells co‐target two antigens to prevent escape	CD33 and CD123	Preclinical: reduced escape and prolonged survival^99^	Reduces antigen escape; improved coverage	Potential increased toxicity, antigen co‐expression required	Preclinical/phase I
iPSC‐derived CAR‐T	Off‐the‐shelf CAR‐T from iPSC; scalable and uniform	CD19, HER2	Early clinical responses (e.g., FT819:4/15 responders)^85^	Scalable; uniform product; reduced variability	Differentiation challenges; allo‐rejection risk; genomic instability	Phase I
UCAR‐T	Allogeneic γδ T cells with CAR; HLA‐independent killing	CD20, NKG2D ligands	67% ORR in B‐cell malignancies (AD1‐001)^156^	Low GvHD risk; intrinsic tumour sensing	Rare cell source; expansion difficulties	Phase I
Armoured CAR‐T (IL‐12)	CAR‐T secreting IL‐12 to remodel TME and enhance persistence	Tumour‐specific + IL‐12	Preclinical: enhanced efficacy; TME reprogramming^35^, ^36^	Counteracts TME; enhances T‐cell function	Cytokine‐related toxicity (CRS/ICANS); narrow therapeutic window	Early‐phase trials
Logic‐gated CAR‐T	AND/NOT‐gates improve tumour specificity and safety	MSLN + CDH3; CD33 + CD123	Preclinical: reduced on‐target, off‐tumour toxicity^101^, ^108^	Enhanced safety; precision targeting	Design complexity; leaky activation; unproven in humans	Preclinical/phase I
In vivo CAR‐T	CAR gene delivered directly to T cells in vivo	CD19, solid tumour antigens	Mouse models: tumour regression^95^, ^97^	No ex vivo manufacturing; preserves T‐cell fitness	Delivery specificity; safety control; transient expression	Preclinical

Abbreviations: AML, acute myeloid leukaemia; CAR‐DC, chimeric antigen receptor‐engineered dendritic cell; CAR‐T, chimeric antigen receptor T; CLL1, C‐type lectin‐like molecule‐1; CRS, cytokine release syndrome; GvHD, graft‐versus‐host disease; HLA, human leukocyte antigen; ICANS, immune effector cell‐associated neurotoxicity syndrome; IL, interleukin; LSC, leukaemia stem cell; MSLN, mesothelin; ORR, overall response rate; OS, overall survival; TME, tumour microenvironment.

## 6 DESIGN AND FUNCTION OPTIMIZATION OF CAR‐DCS

**FIGURE 2:**
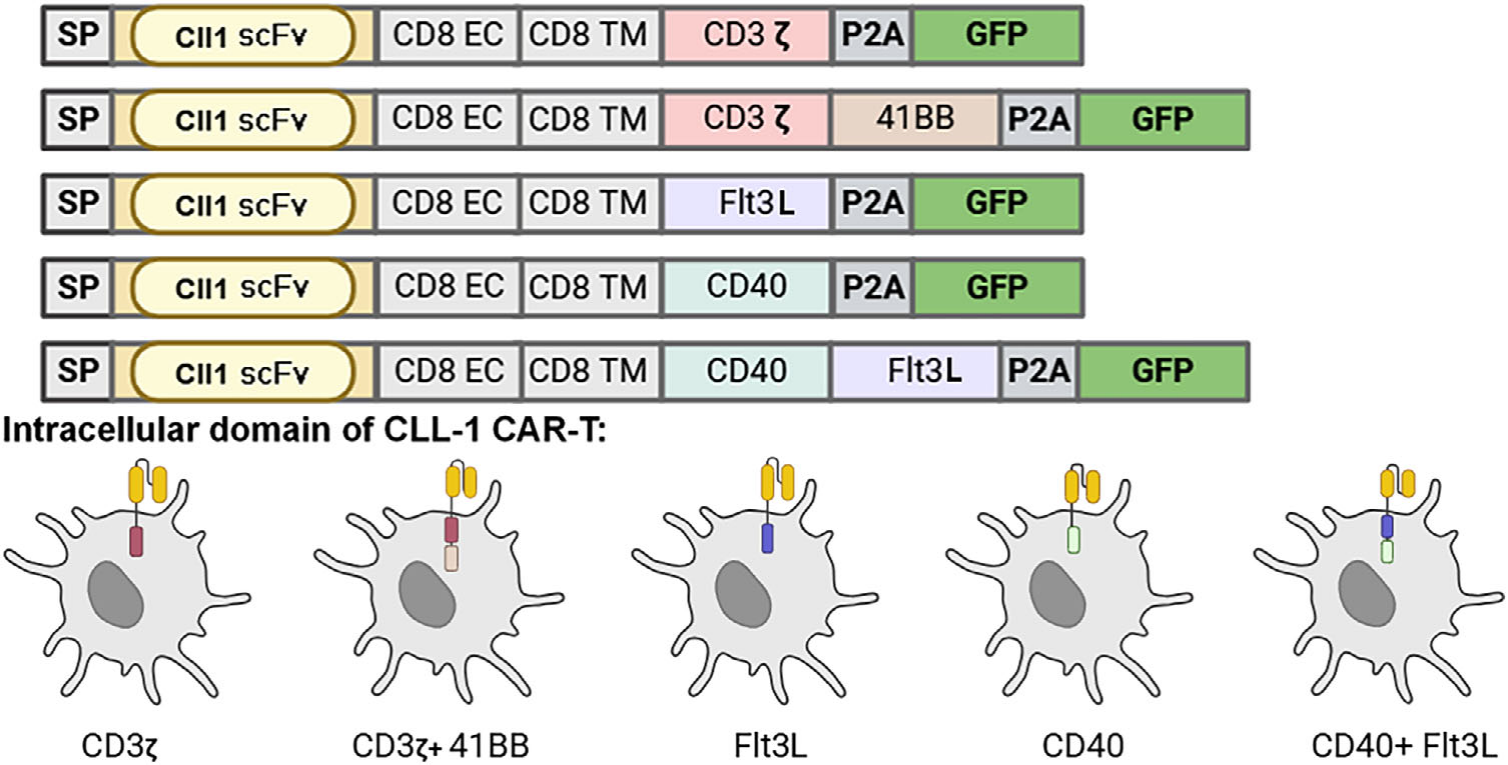
Optimal intracellular signal of chimeric antigen receptor‐engineered dendritic cell (CAR‐DC): selecting the appropriate intracellular domain (CD3ζ; CD3ζ + 4‐1BB; FLT3L; CD40; and CD40 + FLT3L) of CAR can induce the mature phenotype of CAR‐DC.

The selection and design of the intracellular signalling region of CAR is critical in DC‐based therapy, as it enables DC differentiation, phagocytosis and antigen cross‐presentation following tumour antigen recognition (Figure 2):

1. 4‐1BB signalling domain: CD33 CAR‐DCs containing a 4‐1BB‐CD3ζ intracellular region can activate CAR‐T cells and enhance their antitumour activity in AML co‐culture assays.^80^
2. Flt3L signalling domain: the University of Washington developed CAR‐DCs with an FLT3‐integrated domain, to support cDC differentiation and improve cross‐presentation of tumour antigen.^72^ Flt3L‐secreting engineered T cells synergise with pattern recognition receptor poly(I:C) and 4‐1BB agonists to increase intratumoural DC accumulation and systemic antitumour responses, mitigating antigen‐negative tumour escape in solid tumours.^39^, ^75^
3. CD40 signalling domain: CD40 signalling activation increases MHC‐II, co‐stimulatory molecules (CD86/CD70/CD80), and cytokines (IL‐12, TNF‐α), thereby strengthening cross‐presentation.^84^ Preclinical evidence indicates that CD40‐containing CAR‐DCs combined with BCMA CAR‐T cells improve antimyeloma activity.^23^

Thus, appropriate intracellular domain selection (e.g., 4‐1BB, FLT3 or CD40) can promote maturation of CAR‐DCs following tumour encounter and counteract the suppression of the TME.

## 7 EMERGING TECHNOLOGIES AND FUTURE DIRECTIONS

### 7.1 iPSC‐derived allogeneic CAR products

iPSC‐derived allogeneic CAR platforms provide an off‐the‐shelf solution to reduce the cost, complexity and variability associated with autologous therapies.^85^ FT819 (anti‐CD19 CAR‐T cells with a TRAC‐integrated CAR) showed no dose‐limiting toxicity, GVHD or severe CRS in a phase I trial, with responses in four of 15 patients, FT825/ONO‐8250 (anti‐HER2, seven edits) seeks to improve solid tumour targeting. iCAR‐NK programs, including FT596 (anti‐CD19 + hnCD16 + IL‐15RF), demonstrated responses in nine out of 17 patients, while FT522/FT576 include edits for durability and combination therapy. Century therapeutics CNTY‐101, a multi‐edited CD19‐targeting iCAR‐NK therapy, showed no significant toxicity and a 40% response rate in phase I. These findings demonstrate the potential for standardised, scalable and allogeneic immunotherapies.^86^ The core advantage of iPSC platforms lies in generating unlimited, uniform CAR‐DCs and CAR‐T cells to overcome cost and manufacturing barriers inherent to autologous dual‐cell therapy.

The development of functionally mature iPSC‐derived CAR‐T (iCAR‐T) cells encounters significant technical constraints. Current differentiation methods primarily produce innate‐like T cells (iT cells) expressing CD8αα and CD56 but lacking CD8αβ, CD2, CD5 and CD28.^87^, ^88^, ^89^, ^90^ A major issue is inefficient progression through the double‐positive (DP) stage, essential for conventional αβ T‐cell development. Premature TCR or CAR expression, commonly observed with T‐cell‐derived iPSCs or constitutive CAR constructs, disrupts DP formation and biases differentiation towards innate CD8αα⁺ or γδ T‐like phenotypes by suppressing Notch signalling (reduced Notch1/3 and downstream effectors) and decreasing PTCRA expression, which is necessary for αβ T‐cell maturation.^89^, ^91^

Strategies to improve T‐lineage commitment include (a) removing double‐negative T cells to avoid DP progenitor depletion; (b) using OP9‐DLL4 or 3D thymic organoids to increase Notch signalling; (c) employing CAR designs with 4‐1BB instead of CD28 to enhance DP transition and CD8αβ⁺ development; (d) targeting TRAC insertion with attenuated ITAMs to limit tonic signalling and preserve Notch/PTCRA expression; and (e) supplying CAR antigen and 4‐1BBL during differentiation to support DP‐to‐SP maturation. Most systems predominantly yield CD8⁺ iCAR‐T cells, even when CD4⁺ starting populations are used. Co‐culture with artificial thymic organoids enables CD4⁺ iT‐cell production, and a recent feeder‐free approach using PMA/ionomycin to bias DP cells towards the CD4⁺ lineage offers a scalable alternative. Balanced CD4⁺/CD8⁺ ratios may improve persistence and antitumour efficacy.

These findings highlight the difficulty of dual genetic engineering (e.g., incorporating a CAR while maintaining DC differentiation potential in iPSCs) and underscore the necessity of eliminating alloreactivity, such as through human leukocyte antigen (HLA) editing, which further increases developmental complexity.

### 7.2 In vivo CAR cell programming

Direct in vivo CAR gene delivery can intrinsically activate T cells, for example, via innate immune sensing triggered by RNA in LNPs. Unlike strong artificial stimulation applied during ex vivo manufacturing, in vivo‐generated CAR‐T cells expand gradually under physiological antigen drive, preserve stem‐like characteristics, avoid exhaustion and exhibit sustained antitumour activity.^92^ In vivo delivery may preferentially transduce less‐differentiated T cells, including naïve (Tn), stem‐like memory or central memory subsets, which possess superior proliferative capacity, persistence and antitumour potency. Reprogramming even a small number of these cells can result in durable responses. Local administration (e.g., intratumoural) can further enhance efficacy by engineering tissue‐resident memory T cells, enabling direct tumour targeting without reliance on trafficking.^93^ Dynamic TME activation: in vivo‐generated CAR‐T cells undergo progressive activation within the TME. Synergy with the intact host immune system: because in vivo CAR‐T therapy often requires none or only limited lymphodepletion, host immunity remains largely preserved.^94^

Conventional ex vivo CAR‐T‐cell products require complex, multi‐week manufacturing and are constrained by variability in patient T‐cell fitness. In vivo CAR‐T therapy instead administers CAR‐encoding vectors directly to patients. Two major systems are used: viral vectors (e.g., AAV and lentivirus), which support prolonged expression without integration but lack intrinsic T‐cell tropism. Lentiviruses can be pseudotyped with targeting ligands (e.g., anti‐CD3/CD8 scFv) to improve T‐cell specificity. Successful in vivo CAR‐T generation has eliminated CD19^+^ B‐cell malignancies in preclinical studies.^95^, ^96^ Challenges include insertional mutagenesis, off‐target transduction (e.g., germline or malignant clones) and pre‐existing immunity.

### 7.3 Non‐viral nanoparticle platforms

LNPs or polymer‐based nanocarriers primarily deliver CAR‐encoding mRNA for transient expression or DNA for potential stable integration (e.g., piggyBac transposon). Untargeted LNPs accumulate mainly in the liver and spleen, whereas targeted LNPs conjugated with T‐cell‐specific antibodies (e.g., anti‐CD5) improve delivery accuracy. Polymer nanoparticles encoding CAR mRNA against prostate or liver tumour antigens inhibited tumour growth in mice.^97^ AAV‐mediated in vivo CAR‐T production induced tumour regression in murine models.^98^ Loop33 and 123 CAR‐T cells targeting CD33 and CD123 eliminated AML cells and prolonged survival in leukaemia‐bearing mice, while mitigating immune escape.^99^ Key challenges are transient expression, requiring repeated dosing, as well as the increased manufacturing complexity and potential immunogenicity associated with antibody conjugation.

Key limitations include: (1) delivery efficiency and specificity—achieving targeted gene transfer exclusively into desired T‐cell subsets remains difficult; (2) safety control—unlike ex vivo products, in vivo‐generated CAR‐T cells cannot undergo batch‐level phenotyping or safety‐switch activation prior to infusion; (3) transient expression risk—non‐viral systems may yield short‐lived CAR expression, necessitating repeat administration.

### 7.4 Logic‐gated and synthetic gene circuits

Boolean logic gates (e.g., ‘OR’, ‘AND’ and ‘NOT’) represent recent innovations for enhancing CAR‐T precision and safety.

The simplest AND‐gate strategy targets two tumour‐associate antigens (TAAs) co‐expressed on cancer cells but not normal cells, enabling cytotoxicity only when both antigens are engaged.^100^ For TCEs, selective elimination of dual‐TAA⁺ cells results from cooperative binding (pseudo‐avidity).^101^ A trispecific TCE against B7‐H4 and LY6E demonstrated preferential activity in colorectal cancer.^102^ ISB2001 (anti‐BCMA/CD38) functions as a strict AND‐gate, showing a 100‐fold activity reduction after single‐antigen deletion.^103^ Split haemibody TCEs position anti‐CD3 VH/VL domains on separate proteins that reassemble only on dual‐TAA⁺ cells.^104^ The precision GATE platform incorporates extended‐half‐life haemibodies and protease masks to improve performance.^105^, ^106^ Split CAR systems segregate activation and co‐stimulation signals. A low‐affinity CD138 (stimulatory) CAR paired with a high‐affinity CD38 (co‐stimulatory) CAR distinguished myeloma tissue from healthy tissue.^107^ Tousley et al. developed a leak‐resistant AND‐gate CAR incorporating LAT and SLP76 domains to require dual antigen input.^108^ SynNotch CARs, such as anti‐EGFRvIII sensors driving EphA2/IL13Rα2‐CAR expression, reduce antigen escape in glioblastoma^109^ and are in clinical evaluation (NCT06186401).

OR‐gate designs incorporate two or more binders into a single CAR^110^, ^111^, ^112^ or express multiple mono‐specific CARs within a single vector.^113^, ^114^, ^115^, ^116^ Bicistronic CARs allow distinct co‐stimulatory domains per construct to broaden signalling diversity.^117^, ^118^ Careful antigen selection ensures binding to one target does not compromise recognition of the other, particularly in tandem designs.^111^ Dual/triple antigen targeting has been widely applied in B‐cell lymphomas,^119^, ^120^ where antigen loss drives relapse.^121^ Since B‐cell aplasia is manageable clinically, simultaneous targeting of CD19, CD20 and CD22 is common.^122^, ^123^ Tian et al. developed a bicistronic CAR against GPC2 and B7‐H3 for neuroblastoma,^124^ addressing intratumoural heterogenicity. CITE‐Seq identified optimal CARs for T‐cell expansion and phenotype. Compared with single‐target CAR‐T cells, the bicistronic product eliminated single‐ and dual‐positive tumour cells in vitro and in vivo, and demonstrated enhanced persistence and reduced exhaustion in mixed xenografts. An alternative strategy engineers CAR‐T cells to secrete bispecific TCEs recognising a second antigen, creating a localised OR‐gate.^125^

NOT‐gate logic relies on differential expression of a protective antigen present on normal cells but absent on tumour cells, analogous to NK‐cell inhibition via KIR receptors.^126^ Inhibitory CARs (iCARs) contain ITIM motifs that suppress T‐cell activation upon binding to a protective antigen. Early iCARs incorporating PD‐1 or CTLA‐4 intracellular domains enabled rapid, reversible inhibition, outperforming irreversible kill switches.^127^, ^128^ Enhancing iCAR avidity and incorporating dual‐inhibitory domains (e.g., PD‐1 combined with LAIR‐1 or SIGLEC‐9) substantially improves Boolean precision and reduces escape signalling latency.^129^ Key design considerations include iCAR avidity and expression relative to the activating CAR. Increasing affinity alone may not enhance inhibition, whereas increased avidity, such as through higher receptor density or target abundance, can markedly improve suppression.^130^ Notably, iCARs often inhibit proliferation and cytokine release more efficiently than immediate cytotoxicity, which depends on pre‐formed granules.^131^

A clinically promising strategy leverages tumour HLA loss of heterozygosity. The Tmod system pairs an inhibitory CAR recognising HLA‐A*02 (commonly lost in tumours) with an activating CAR targeting an antigen such as mesothelin or carcinoembryonic antigen.^132^, ^133^ Tumour cells that lose β2‐microglobulin (β2M) evade inhibitory signalling and remain susceptible to killing, whereas normal HLA‐A02^+^/β2M⁺ cells are protected.^134^ Our group developed CD16‐CLL1 iCAR‐T cells that retain activity against leukaemia while sparing neutrophils, significantly reducing the incidence of granulocytopaenia during CAR‐T‐cell therapy.^135^

Limitations include: (1) engineering complexity and off‐target risk—complex multi‐antigen logic designs (e.g., tandem CARs, split circuits) may cause scFv interference or ‘leaky’ signalling, increasing on‐target/off‐tumour toxicity. (2) Strict antigen‐profile requirements—AND‐gates require co‐expression of two tumour‐restricted antigens, whereas NOT‐gates depend on consistent absence of a protective marker on malignancies. Tumour heterogeneity and antigen loss remain major escape routes. (3) Kinetic and potency constraints—inhibitory signals may be too slow to prevent early cytotoxic damage to normal cells, and intricate signalling modules may reduce overall T‐cell vigor. (4) Limited clinical validation—most logic‐gated systems remain in preclinical or early clinical phases. Their performance in human TMEs is not definitively established and suppressive tumour niches may impair circuit fidelity.

Armoured CAR‐T designs aim to enhance antitumour function by enabling autocrine cytokine support. IL‐2: IL‐2 secretion promotes T‐cell proliferation and tumour regression in preclinical melanoma models,^136^, ^137^, ^138^ but clinical trials revealed toxicity without efficacy improvement.^139^ IL‐12: IL‐12 is highly potent yet systemically toxic.^140^ Tumour‐localised IL‐12 from CAR‐T cells increases antitumour immunity and reprograms myeloid compartments in murine models.^141^, ^142^, ^143^ Inducible systems (e.g., NFAT‐regulated IL‐12) improve safety,^144^, ^145^ and CD19‐CAR/IL‐12 T cells achieved tumour clearance and resistance to Treg‐mediated suppression in syngeneic settings.^146^ IL‐15: IL‐15 enhances T‐ and NK‐cell persistence^147^, ^148^, ^149^; constitutive expression improves CAR‐T durability in preclinical studies,^150^, ^151^ yet uncontrolled proliferation and leukemogenic events have been reported.^152^ Incorporating inducible suicide switches (e.g., iCaspase9) improves safety.^153^ IL‐21: ex vivo IL‐21 improves expansion and cytotoxicity of CAR‐T cells against Nalm6,^154^ although the feasibility of IL‐21 transgenes requires further assessment.

Limitations of armoured CARs include: (1) cytokine‐linked toxicity—even restricted IL‐12 or IL‐15 secretion risks CRS and ICANS. (2) Control challenges—tight temporal regulation is difficult; constitutive cytokine production can drive uncontrolled proliferation and malignant transformation. (3) Narrow therapeutic index—clinical experience, especially with IL‐2, shows toxicity often rises faster than benefit. (4) Limited long‐term clinical evidence—safety and durability of inducible cytokine‐engineering systems remain insufficiently tested in large trials.

Multiple cell types for UCAR‐T‐cell manufacturing show natural resistance to graft‐versus‐host disease (GvHD). These include γδ T cells, invariant natural killer T cells (iNKTs), double‐negative T cells (DNTs) and virus‐specific T cells (VSTs), alongside engineered platforms such as iPSCs and placental circulating T (P‐T) cells. Their distinctive antigen‐recognition mechanisms offer potential advantages in solid tumours.

γδ T cells: γδ T cells (5% of peripheral CD3⁺ cells, dominated by Vγ9Vδ2) recognise ligands independent of HLA, minimising GvHD risk.^155^ Their innate cytotoxicity persists even after CAR‐target antigen loss, addressing heterogeneity.^104^ ADI‐001 (CD20‐targeted γδ UCAR‐T) achieved a 67% overall response rate in B‐cell malignancies, and dose‐dependent expansion despite HLA mismatch.^156^, ^157^ γδ T cells also exploit NKG2D‐mediated targeting, for instance, temozolomide upregulates NKG2DL in glioblastoma, enhancing γδ T‐cell tumour recognition.^158^

iNKT cells: iNKT cells recognise glycolipids via CD1d, an HLA‐independent mechanism that reduces GvHD risk.^159^ They preferentially home to tumours through chemokines such as CCL2 and CCL20, making them advantageous for solid tumour targeting.^160^ CAR‐iNKTs have been investigated in both haematologic and solid malignancies.^161^, ^162^ An advanced allogeneic CD19‐CAR iNKT product co‐expressing IL‐15 and shRNAs against B2M/CD74 (to reduce HLA‐I/II expression) demonstrated efficacy in R/R non‐hodgkin lymphoma and acute lymphoblastic leukemia.^163^ Autologous GD2‐CAR iNKTs expressing IL‐15 induced a complete response in neuroblastoma.^164^ Beyond direct tumour killing, CAR‐iNKTs contribute to host immunity by cross‐priming CD8⁺ T cells,^107^ depleting immunosuppressive CD1d⁺ TAMs and MDSCs,^165^, ^166^ activating DCs, and promoting epitope spreading.^108^

DNTs: DNTs (CD3⁺CD4^−^CD8^−^) rarely cause GvHD. Allogeneic DNTs expanded from AML patients showed safety and antitumour activity via NKG2D and DNAM‐1 pathways.^109^, ^167^, ^168^, ^169^, ^170^ CD19‐CAR‐DNTs effectively targeted B‐cell leukaemia and lung cancer without inducing GvHD.^171^ CAR4‐DNTs targeting T‐cell malignancies demonstrated improved persistence with idelalisib.^172^ A phase I trial of allogeneic CD19‐CAR‐DNTs (RJMty19) in B‐cell lymphoma reported no ≥G3 CRS, ICANS, GvHD or DLTs and all high‐dose participants achieved responses.^173^

VSTs and engineered cell sources: VSTs have a restricted TCR repertoire, lowering GvHD risk.^174^, ^175^ They are clinically used to treat post‐HSCT viral infections,^176^ although their cancer applications remain limited.^177^, ^178^ iPSCs offer a renewable CAR‐T source, CAR‐iPSCs differentiated via 3D organoids generate functional T cells with uniform TCRs and reduced MHC expression, minimising GvHD and rejection.^179^ Inhibiting G9a/GLP enhances iPSC‐T‐cell maturation and CAR effector function.^180^

Limitations of allogeneic UCAR‐T‐cell sources: (1) cell source scarcity and expansion difficulty—γδ T cells, iNKTs and DNTs are rare in peripheral blood, increasing the complexity and cost of isolation, engineering and large‐scale expansion. (2) Uncertain persistence and potency—some subsets, such as VSTs, may have limited in vivo persistence and expansion, reducing long‐term activity. (3) Allo‐rejection—host immunity may eliminate allogeneic CAR‐T cells due to HLA mismatch, limiting persistence. Although HLA editing in iPSCs can mitigate this, residual risk remains. (4) Technical complexity—iPSC‐derived CAR‐T manufacturing requires reprogramming, gene editing and controlled differentiation, posing challenges for scalable and consistent production. (5) Unconventional safety profiles—native reactivity of γδ T cells or iNKT may cause on‐target, off‐tumour effects in normal tissues and requires careful evaluation.

## 8 CURRENT CHALLENGES AND LIMITATIONS RELATED TO CAR‐DCS

### 8.1 Plasticity of DCs and intrinsic dysfunction in AML

AML disrupts normal DC development, characterised by an accumulation of arrested DC precursors (Lin^−^HLA‐DR⁺CD11c⁺CD123⁺) and a deficiency of terminal DC subsets (BDCA‐1⁺/BDCA‐3⁺mDCs; BDCA‐2⁺pDCs) in FLT3‐ITD⁺ patients at diagnosis. Impaired myeloid DC function persists even in remission,^181^ suggesting intrinsic maturation defects that may limit CAR‐DC efficacy. Chemotherapeutic agents such as daunorubicin can exacerbate immunosuppression by inducing ATP release from dying blasts, activating the P2X7‐IDO1 axis in DCs and promoting Treg expansion.^182^ AML‐educated DCs and CAR‐DCs may therefore retain aberrant plasticity or tolergenic traits.

Tumour‐induced tolerogenic DC phenotypes: (1) metabolite‐driven tolerance—mregDCs migrate to tumour dLNs, where they suppress cross‐presentation and induce Th2 and Treg differentiation. Tumour‐derived lactate activates sterol regulatory element‐binding protein 2 (SREBP2) in DCs, promoting mevalonate‐dependent mregDC differentiation that suppresses CD8⁺ T cells and enhances Th2/Treg responses.^64^ (2) Stromal signalling—CAF‐secreted WNT2 inhibits DC maturation via SOCS3/p‐JAK2/p‐STAT3 signalling, weakening antitumour immunity.^65^ These pathways suggest that infused CAR‐DCs may be ‘re‐educated’ by the TME into IDO1⁺CD39⁺DCs or mregDCs reducing synergy with CAR‐T cells. (3) Logistical and biological complexity—dual‐cell manufacturing significantly increases cost and complexity versus single‐cell therapies. Predictive biomarkers remain unclear and TP53/KRAS mutations alone may not identify optimal candidates.

Preclinical evidence indicates several theoretical advantages of CAR‐DCs: (1) antigen acquisition fidelity—cDC vaccines rely on tumour lysates or selected peptides, whereas CAR‐DCs actively locate tumour cells in vivo and acquire a diverse antigen repertoire directly from the patient's TME, a critical feature in heterogeneous diseases such as AML. (2) Overcoming DC dysfunction—given that endogenous DCs in AML are often impaired, engineered CAR‐DCs with activation domains (e.g., CD40 or 4‐1BB) can achieve rapid maturation upon CAR signalling and resist TME suppression more effectively than cDC vaccines. (3) Synergistic immuno‐orchestration—beyond functioning as an improved antigen‐presenting platform, CAR‐DCs act as an in situ immune organisers that support co‐administered CAR‐T cells through cytokine secretion (e.g., IL‐12) and induction of epitope spreading. CAR‐DCs combined with CLL1 CAR‐T cells represent a novel approach to overcoming current efficacy barriers in R/R AML. A deeper mechanistic understanding of CAR‐DC biology will support clinical translation and provide a scientific foundation for integrating CAR‐DCs with CLL1 CAR‐T therapy in refractory or relapsed AML.

CAR‐DCs present several challenges: (1) functional complexity—the efficacy of the indirect ‘bystander’ killing of antigen‐negative tumours depends entirely on efficient cross‐presentation and epitope spreading, which may be suboptimal in immunosuppressive settings. (2) Safety considerations—although the synergy between CAR‐DCs and CAR‐T cells offers a compelling strategy to enhance antitumour immunity, both modalities are engineered for robust immune activation, raising concerns regarding amplified immune‐related toxicities, particularly CRS. Preclinical studies of CAR‐DC and CAR‐T co‐administration have demonstrated increased cytokine release (e.g., IL‐6, IL‐12, IFN‐γ), which could heighten CRS risk. Existing preliminary clinical data (e.g., NCT05631899) have not reported severe CRS although sample sizes remain small. CAR‐DCs may support more controlled activation through localised cytokine release and epitope spreading, potentially lowering systemic toxicity compared to standalone CAR‐T therapy. Nonetheless, future clinical protocols should include stringent monitoring, prophylactic measures (e.g., tocilizumab, corticosteroids), and inducible safety switches such as caspase‐based suicide systems to allow rapid intervention in the event of excessive immune activation. (3) TME resistance—while CAR‐DCs can be engineered to secrete cytokines such as IL‐12, the long‐term maintenance of this function and their ability to resist tolerogenic reprogramming by the suppressive TME remain to be proven. (4) CAR design and patient selection—optimisation of intracellular signalling modules (e.g., FLT3 andCD40) should be prioritised and future studies must establish rational patient selection criteria and incorporate predictive biomarkers, including TME profiling.

The combination of CAR‐DCs and CLL1‐targeted CAR‐T cells constitutes a transformative therapeutic strategy to simultaneously address antigen escape and immunosuppression in patients with R/R AML. CAR‐DCs function as first‐wave ‘immune orchestrators’ by remodelling the TME, thereby facilitating a more effective second‐wave attack by CAR‐T cells. Through synergistic enhancement of antigen presentation and reversal of T‐cell exhaustion, this dual‐cell approach has the potential to induce deeper and more durable remissions. To translate this promising preclinical strategy into clinical practice, several critical areas require further investigation. First, in the engineering of CAR‐DCs, the optimal co‐stimulatory signalling domains—such as CD40 for DC maturation versus FLT3L for expansion—must be determined, along with the feasibility of dual‐targeting CARs. Second, clinical development must establish the safety profile of this combination therapy, particularly with respect to CRS, and optimise dosing regimens and routes of administration. The identification of predictive biomarkers will be essential for appropriate patient selection. Furthermore, the therapeutic efficacy may be enhanced through triple‐combination approaches, such as incorporating ICIs to prevent functional exhaustion of both endogenous and CAR‐T cells. While the path from a compelling preclinical concept to an established clinical therapy remains complex, the rationally designed synergy between these two cellular immunotherapies represents a promising advance for improving outcomes in R/R AML.

## AUTHOR CONTRIBUTIONS

*Conceptualisation of the project*: Rui Zhang, Hongkai Zhang and Mingfeng Zhao. *Investigation*: Rui Zhang. *Writing articles and revision of the manuscript*: Rui Zhang and Jinlin Zhang. All the authors approved the final manuscript as submitted and agree to be accountable for all aspects of the work.

## CONFLICT OF INTEREST STATEMENT

The authors declare no potential conflicts of interest concerning the research, authorship and/or publication of this article.

## ETHICS STATEMENT

Ethics approval was provided from the Department of Hematology at Tianjin First Central Hospital (Tianjin, China) (2020N212KY; Tianjin First Central Hospital Medical Ethics Committee). Clinical trial number: ChiCTR2000041025.

## Supporting information



Supporting Information

Supporting Information

Supporting Information

Supporting Information

## Data Availability

All data generated or analysed during this study are included in this article.
