# Supplementary material for: CAR‐DC combined with CAR‐T therapy for relapsed/refractory acute myeloid leukaemia: Research progress and future perspectives
Source: Clin Transl Med. 2025 Nov 25;15(12):e70536. doi: 10.1002/ctm2.70536 (PMC12647367; doi:10.1002/ctm2.70536)
Supplement: Supplementary file 1 — Supporting Information [file CTM2-15-e70536-s004.pdf]

Feng

by orhiwi muroma

Submission date: 17-Aug-2025 08:13PM (UTC+0700)
Submission ID: 2730732957
File name: 查重.docx (31.4K)
Word count: 2292
Character count: 15031

Abtract

Acute myeloid leukemia (AML) remains the most common type of leukemia in adults. Despite advances in conventional therapies, high relapse rates persist, underscoring the need for novel approaches such as Chimeric Antigen Receptor T (CAR-T) cell therapy. CLL1-targeted CAR-T emerges as a promising treatment for relapsed/refractory (R/R) AML. Although approximately 70% patients achieved remission, only a subset achieved minimal residual disease (MRD) negative remission, which still has much room for improvement. The main reasons for the failure of CLL1 CAR-T cell therapy include: (1) Persistence of CLL1-negative AML cells persist due to antigen escape; (2) Downregulation of IL-12 and other cytokines by the immunosuppressive tumor microenvironment (TME) , contributing to the exhaustion of both endogenous T cells and CLL1 CAR-T cells.

We synthesize a combination of CAR-engineered dendritic cells (CAR-DCss) and CLL1 CAR-T cells to overcome current limitations. CAR-DCss enhance antigen cross-presentation to activate endogenous T cells against antigen-negative clones, secrete immunostimulatory cytokines (e.g., IL-12) to sustain CAR-T activity, and remodel the TME. Key challenges involve optimizing CAR designs (e.g., incorporating Fms-like tyrosine kinase 3 ligand (FLT-3L) or CD40 signaling domains), mitigating toxicity, and establishing clinical administration protocols. Collaborative efforts will be critical to translate this synergistic approach into clinical practice.

Keywords: CLL1 CAR-T; CAR-DCs; Acute myeloid leukemia;

1. The current research status of AML treatment

The conventional treatment of Acute myeloid leukemia (AML) mainly includes chemotherapy, targeted therapy and hematopoietic stem cell transplantation, but most patients face the risk of relapse[1-2]. Chimeric Antigen Receptor T (CAR-T) therapy represents a promising novel approach for relapsed/refractory (R/R) AML, although significant challenges remain. Currently, CAR-T cell therapy has achieved impressive clinical outcomes in hematologic malignancies, including acute lymphoblastic leukemia, lymphoma, and multiple myeloma, by targeting CD19, CD22, and BCMA[3-6]. However, CAR-T cell therapy remains clinically immature for AML, with

limited overall efficacy in current studies[7-8]. The primary reasons include the lack of specific targets[9-10], the immunosuppressive tumor microenvironment (TME)[11-12], and antigen escape[13-15]. Early CAR-T therapies for AML targeted CD33 and CD123, but their toxicity or poor efficacy has limited their clinical application[7,16-17]. Other potential targets include CLL1, FLT3, NKG2D, CD7 and CD38, etc[18-23]. CAR-T cells targeting CLL1 exhibit therapeutic potential in patients with R/R AML, and holds significant clinical potential.

2. Therapeutic Efficacy of CLL1 CAR-T Cells in Relapsed/Refractory AML

CLL1 (C-type lectin-like molecule-1) is a C-type lectin-like receptor that is highly expressed on leukemia stem cells (LSCs) (about 45%) and leukemia progenitor cells (77.5-92%). Tashiro et al. developed the first CLL1 CAR-T cells, which selectively kill leukemic progenitor cells and leukemia cells[24]. CLL1 CAR-T cells have shown superior AML killing in vitro and in mice[18]. A case report described complete remission in a 10-year-old patient after CLL1 CAR-T therapy[25]. Our group reported a 70% complete remission rate in 10 adult AML patients treated with CLL1 CAR-T cells[26]. At present, more than 54 patients with refractory or relapsed AML have been treated with CLL1 CAR-T cell therapy, achieving a 73% complete remission rate[27].

Although CLL1-targeted CAR-T cells have shown clinical efficacy in R/R AML, approximately 30% of patients exhibit primary treatment resistance. Furthermore, only 50% of responders achieve minimal residual disease (MRD)-negative remission, underscoring the need for enhanced therapeutic strategies.

Key limitations include: (1) Antigen escape: AML cells with low expression of CLL1 were difficult to be recognized by CAR-T, and relapsed patients often show weak expression of target antigen[28-29]. (2) TME and IL-12: The TME inhibits CAR-T cell efficacy via myeloid-derived suppressor cells (MDSCs), regulatory T cells (Tregs) and tumor-associated macrophages (TAMs)[30-32]. Additionally, dysregulated cytokine levels (e.g., IL-12) impair CAR-T cells function. mKRAS-specific NeoCARs with inducible IL-12 secretion and T cell receptor (TCR) knockout demonstrate potent in vivo antitumor activity and a favorable safety profile[33]. IL-12 and IFNα2 co-expression with CAR augments proinflammatory TME and mitigates T-cell

exhaustion[34]. mbIL12 -engineered CAR T cells proved safe and effective, overcoming the TME [35]. IL-12 not only enhances CAR-T cell cytotoxicity but also reprograms TME, increasing proinflammatory $CD4^+$ T cell infiltration, reducing regulatory T cells (Tregs), and activating the myeloid compartment. Critically, these immunotherapy-enabling effects were achieved with minimal systemic toxicity in GBM-targeted CAR-T cell therapy[36]. Therefore, combined strategies targeting both CAR-T cell engineering and TME modification are essential to enhance the clinical effectiveness of CLL1 CAR-T therapy.

3. Research progress and challenges of DC cell therapy

Dendritic cell (DC) mediate antitumor immunity by phagocytosing tumor material, processing tumor antigens, and presenting peptide-MHC complexes to activate tumor-specific T cells[37-38]. Concomitantly, mature DC cells engage directly with T cells through costimulatory molecules (CD80/CD86) and secrete IL-12, and activate the priming of endogenous tumor-specific T cells[39-41].

DC therapy primes tumor-reactive T cells by capturing, processing, and presenting tumor antigens through MHC molecules. It's reported that mature DC initiated tumor-specific $CD8^+$ T cell immunity by migrating from tumors to lymph nodes, capturing antigens, and activating naïve T cells[42]. Conventional dendritic cells (cDCs) are broadly categorized into two functionally distinct subsets: type 1 (cDC1) and type 2 (cDC2), which exhibit distinct functional roles[43]. cDC1s prime $CD8^+$ T cells in specific regions of the draining lymph nodes (dLNs). They activate Toll-like receptors (TLRs) to secrete IL-12p70 and IFN-α, driving Th1-type immunity. Due to their ability to stimulate anti-tumor immunity, cDC1s serve as a favorable prognostic marker in cancer patients[44]. cDC2s, which are more abundant than cDC1s, express surface markers such as CD11c, CSF-1R, MHC-II, CD11b, BDCA1 and SIRPα. Human cDC2s produce a variety of cytokines, including IL-23 and IL-10, and present antigens to $CD4^+$ helper T cells, thereby activating effector T-cell subsets such as Th2 and Th17 cells[45]. In vivo, cDC1 are uniquely capable of tumor antigen cross-presentation and essential for generating adaptive antitumor immunity; without cDC1, tumors evade

immune elimination[46]. In AML patients, the frequency and function of dendritic cells are often impaired, contributing to disease progression and therapy resistance[47]. Given their exceptional capacity for T-cell activation and cross-presentation, cDC1s are a preferred subset in CAR-DCs-based immunotherapies.

DC/tumor fusion vaccines or tumor lysates loaded on DC cells can mediate the activation and persistence of anti-tumor T cells, and expand the clonal population of T cells targeting tumor cells[48]. Recently, a phase II clinical trial (NCT03059485) reported that DC/AML fusion vaccination, without maintenance therapy, achieved a 73% 2-year overall survival (OS) and 36% progression-free survival (PFS) in elderly AML patients[49]. Eps8-DCs boosted CD19 CAR-T cell functions, augmenting cytokine secretion, CD107a degranulation and cytotoxic activity[50]. However, DC dysfunction contributes to immune evasion and limits efficacy of DC vaccines in elderly patients[51]. The clinical efficacy of moDC vaccines remains variable, limited by challenges in antigen loading, incomplete maturation, and tumor heterogeneity. It has been reported that the secretion of IL-12 directly affects the function of endogenous DC1[49,52-53] .

TME impairs DC function through multiple inhibitory mechanisms, including: (1) Induction of DC apoptosis[54-55]; (2) Suppression of DC maturation and antigen presentation[56-57]; (3) Promotion of tolerogenic DC phenotypes[58-59] ; (4) Downregulation of DC-recruiting chemokines to limit tumor infiltration[60-63].

Further improvements include the induction of DC reprogramming. The DC growth factor Fms-like tyrosine kinase 3 ligand (Flt3L) enhances T cell-mediated anti-tumor immunity by expanding and activating DC populations[46]. It has been reported that highly activated DC can induce $CD4^{+}$T cells to acquire cytotoxic and anti-tumor functions in aged mice[50]. PU.1, BATF3 and IRF8 mediated DC reprogramming reduced exhaustion and increased memory and stem cell-like T cell infiltration[64]. Knockout of BCL9/BCL9L by cDC1 enhanced $CD8^{+}$T activation, antigen presentation and epitope expansion, and enhanced anti-tumor activity[65-66].

4. Research progress and challenges of CAR-DCs cell therapy

CAR-DCss can specifically recognize tumor antigens, efficiently phagocytose CAR-targeted tumor cells and debris, and subsequently activate endogenous tumor-specific T cell responses. Antitumor T-cell responses can directly eliminate CAR$^-$-targeted antigen-positive tumors and indirectly eliminate CAR tumor cells (which are not directly recognized by the CAR) through cross-presentation and epitope spreading[61,67]. Activated CAR-DCs cells can secrete immunostimulatory cytokines (e.g., IL-12 that may help counteract the immunosuppressive TME and enhance immune cell function within tumors[35,61,68-69]. The intracellular signal transduction domain of CAR can be continuously activated during tumor recognition, which allows DC to present a mature homeostasis and overcome the microenvironment, leading to the acclimation of tolerant DC.

5. **CAR-DCs cells Combined with CAR-T cells Therapy**

CAR-DCs have been demonstrated effective against acute myeloid leukemia in preclinical studies and are being studied in human against solid epithelial malignancies (NCT05631899 & NCT05631886). NCT05631899 is a pilot clinical trial evaluating the safety, immune response and efficacy of an EphA2-targeting CAR-DCs vaccine loaded with KRAS mutant peptide (KRAS-EphA2-CAR-DCs) in combination with immune checkpoint inhibitors (ICIs) for patients with locally advanced or metastatic solid tumors. Preclinical studies demonstrated that these engineered CAR-DCs enhance the cytotoxicity of co-administered CAR-T cells in solid tumor mouse models. NCT05631886 is a parallel pilot trial investigating a similar EphA2-directed CAR-DCs vaccine with TP53 mutant peptide (TP53-EphA2-CAR-DCs) plus ICIs, enrolling patients with solid tumors or R/R lymphomas. Emerging evidence suggests that intratumoral delivery of DCs can safely potentiate CAR-T cell activity and improve the immunosuppressive TME. The study demonstrated that in vitro differentiation of DCs expressing 4-1BB enriches the CD141$^+$/Clec9A$^+$ DC subset. Furthermore, CAR-DCs and CAR-T cell interactions synergistically enhance anti-AML cytotoxicity[70]. DC-derived cytokines (e.g., IL-12 and type I IFNs) provide additional T cell stimulation during antigen presentation, while CAR-T cells retain intrinsic tumor-killing capacity independent of DC involvement[71]. These results showed

intratumoral CAR-DCs delivery creates an "immunological niche". CAR-DCs secrete IL-12 and Type I IFNs reverse T-cell exhaustion and TEM. The synergy hinges on overcoming CAR-T limitations (poor infiltration, antigen loss) through DC-mediated antigen spreading and localized immune activation. Studies demonstrate that 4-1BB-engineered autologous DCs enhance the efficacy of anti-CD33 CAR-T therapy in AML by secreting cytokines and promoting CAR-T cell recruitment to the bone marrow niche[71]. The synergistic interaction between CAR-DCs and CAR-T cells may provide a novel strategy to boost antitumor efficacy in cancer immunotherapy.

Mechanistic overview of CAR-DCs cells and CAR-T cells collaboration (Figure 1) : (1) CAR-DCs cells phagocytoses AML cells via CAR targeting, processes antigens, and cross-presents tumor-derived peptides via MHC-I/II to activate endogenous T cells[40-41]. (2) CAR-DCs secretes cytokines (e.g., IL-12) to support the activation, proliferation, and persistence of CAR-T cells, potentially reducing exhaustion and promoting a memory phenotype[40,35,71,73]. (3) Activated endogenous T cells (primed by CAR-DCs) and CAR-T cells work together to eliminate both CLL1-positive and CLL1-negative (via epitope spreading) AML clones[74-75].

Therefore, CAR-DCs and CAR-T therapy have a good complementarity, and have the potential to enhance the function of CAR-T and overcome immune escape to avoid tumor recurrence.

6. Design and function optimization of CAR-DCs

The selection and design of the intracellular signal transduction region of CAR is the key in DC cell therapy. It has the ability to promote DC cell differentiation, phagocytosis and/or antigen cross-presentation after tumor antigen recognition by CAR. Current Research (Figure2):

(1) 4-1BB Signaling Domain: CD33 CAR-DC cells with a 4-1BB-CD3ζ intracellular domain have been shown to activate CAR-T cells and enhance their antitumor efficacy in AML when co-cultured in vitro[72].

(2) Flt3L Signaling Domain: The University of Washington engineered CAR-DCs cells with an FLT3-integrated intracellular domain, enabling cDC differentiation and enhanced cross-presentation of tumor antigens[64]. Flt3L-secreting engineered T cells

synergize with pattern recognition receptor (poly(I:C)) and co-stimulatory (anti-4-1BB) agonists to enhance intratumoral DC accumulation and systemic antitumor immunity. This approach mitigates antigen-negative tumor escape in solid tumors[39, 67].

(3) CD40 Signaling Domain: The CD40 signaling cascade upregulates MHC class II, costimulatory molecules (CD86/CD70/CD80), and cytokines (IL-12, TNF-α), thereby potentiating antigen cross-presentation[76]. Preclinical studies demonstrate that CAR-DCs cells with a CD40 intracellular domain, when combined with BCMA CAR-T cells, improve multiple myeloma treatment efficacy[23].

Therefore, selecting the appropriate intracellular domain (e.g., 4-1BB, FLT3, or CD40) of CAR can induce the mature phenotype of CAR-DCs when recognizing the tumor, and overcome the acclimation of tolerant DC cells by the suppressive tumor microenvironment.

7. Conclusions and Prospects

CAR-DCs combined with CLL1 CAR-T provides a new strategy to break through the bottleneck of the existing efficacy for R/R AML. An in-depth understanding of the mechanism of CAR-DCs will contribute to the clinical transformation of CAR-DCs and provide a scientific basis for the combination of CAR-DCs and CLL1 CAR-T in the treatment of refractory or relapsed AML patients. Challenges in clinical translation:

（1）Safety: Dual-CAR risks exacerbating CRS/neurotoxicity (needs phased clinical evaluation)

（2）Administration: Optimal dosing/sequencing of CAR-DCs and CAR-T requires validation

（3）CAR Design: FLT3/CD40 signaling domains warrant prioritization

Future work should define patient selection criteria and integrate biomarkers (e.g., TME profiling).

8. Current Challenges and Limitations

DC Plasticity and Intrinsic Dysfunction in AML: AML intrinsically subverts DC differentiation, as evidenced by the predominance of arrested DC precursors ($Lin^{-}HLA\text{-}DR^{+}CD11c^{+}CD123^{+}$) and deficiency in terminal DC subsets (BDCA-1^{+}

/BDCA-3^{+}mDCs; BDCA-2^{+}pDCs) in FLT3-ITD^{+}patients at diagnosis. Critically, myeloid DC impairment persists even in remission[77], suggesting inherent defects in DC maturation that may compromise CAR-DCs functionality. Furthermore, conventional chemotherapy (e.g., daunorubicin) exacerbates immunosuppression by inducing ATP release from dying blasts, activating the P2X7-IDO1 axis in DCs to drive Treg expansion and tolerogenesis[78]. This raises concerns that AML-educated DCs and CAR-DCss may retain aberrant plasticity or immunosuppressive traits.

Tumor-Induced Tolerogenic DC Phenotypes:

(1) Metabolite-Driven Tolerance: mregDCs exhibit migratory capacity to tumor-draining lymph nodes, where they mediate trans-suppression of DC-mediated antigen cross-presentation. mregDCs promote T helper 2 (Th2) and Treg differentiation. Tumor-secreted lactate induces 19 sterol regulatory element-binding protein 2 (SREBP2) activation in DCs, triggering mevalonate pathway-dependent differentiation into mregDCs that suppress CD8^{+}T cells and promote Th2/Treg responses[56].

(2) Stromal Signaling: CAF-secreted WNT2 inhibits DC differentiation via SOCS3/p-JAK2/p-STAT3 signaling, blunting antitumor immunity[57].

These pathways highlight a critical limitation: infused CAR-DCss may undergo "re-education" by the TME, adopting tolerogenic phenotypes (e.g., IDO1^{+}CD39^{+}DCs or mregDCs) that undermine CAR-T collaboration.

Logistical and Biological Complexity:

(1) Manufacturing challenges: Dual cellular products (CAR-DCs+CAR-T) increase cost/complexity vs. single-agent therapies.

(2) Lack of predictive biomarkers: Unclear which patients benefit most from synergy (e.g., TP53/KRAS mutational status alone may be insufficient).

Feng

ORIGINALITY REPORT

12%	6%	8%	1%
SIMILARITY INDEX	INTERNET SOURCES	PUBLICATIONS	STUDENT PAPERS

PRIMARY SOURCES

1	pmc.ncbi.nlm.nih.gov<br>Internet Source	2%
2	Chunrun Qu, Hao Zhang, Hui Cao, Lanhua Tang et al. "Tumor buster - where will the CAR-T cell therapy ‘missile’ go?", Molecular Cancer, 2022<br>Publication	1%
3	Hui Zhang, Chaoke Bu, Zhiyong Peng, Guangchao Li et al. "Characteristics of anti-CLL1 based CAR-T therapy for children with relapsed or refractory acute myeloid leukemia: the multi-center efficacy and safety interim analysis", Leukemia, 2022<br>Publication	1%
4	www.biomolther.org<br>Internet Source	1%
5	Andy Kah Ping Tay. "CAR-T Manufacturing - Technologies and Innovations", CRC Press, 2025<br>Publication	1%
6	Javed N. Agrewala. "Immunotherapy in Autoimmune Disorders - Mechanisms and Applications", CRC Press, 2026<br>Publication	1%
7	www.kxan.com<br>Internet Source	1%

8	Hui Zhang, Wen-Ting Gan, Wen-Ge Hao, Peng-Fei Wang, Zhuo-Yan Li, Lung-Ji Chang. "Successful Anti-CLL1 CAR T-Cell Therapy in Secondary Acute Myeloid Leukemia", Frontiers in Oncology, 2020<br>Publication	1 %
9	Submitted to University of Birmingham<br>Student Paper	1 %
10	Iqbal S. Grewal. "Emerging Protein Biotherapeutics", CRC Press, 2019<br>Publication	<1 %
11	www.ncbi.nlm.nih.gov<br>Internet Source	<1 %
12	www.onderzoekmetmensen.nl<br>Internet Source	<1 %
13	ijcimr.org<br>Internet Source	<1 %
14	Faroogh Marofi, Heshu Sulaiman Rahman, Zaid Mahdi Jaber Al-Obaidi, Abduladheem Turki Jalil et al. "Novel CAR T therapy is a ray of hope in the treatment of seriously ill AML patients", Stem Cell Research & Therapy, 2021<br>Publication	<1 %
15	Prateek Pophali, Giulia Cheloni, Richard M. Stone, Kai W Wucherpfennig et al. "Randomized Phase II Trial of Dendritic Cell/AML Fusion Cell Vaccination Compared to Standard of Care Therapy in AML CR1", Blood, 2024<br>Publication	<1 %
16	assets-eu.researchsquare.com<br>Internet Source	<1 %

17	www.mypublichealth.pitt.edu<br>Internet Source	<1 %
18	5dok.org<br>Internet Source	<1 %
19	academic.oup.com<br>Internet Source	<1 %
20	patents.google.com<br>Internet Source	<1 %
21	Alessandro Isidori, Claudio Cerchione, Naval Daver, Courtney DiNardo et al. "Immunotherapy in Acute Myeloid Leukemia: Where We Stand", Frontiers in Oncology, 2021<br>Publication	<1 %
22	Hui Zhang, Pengfei Wang, Zhuoyan Li, Yingyi He, Wenting Gan, Hua Jiang. "Anti-CLL1 chimeric antigen receptor T cell therapy in children with relapsed/refractory acute myeloid leukemia", Clinical Cancer Research, 2021<br>Publication	<1 %
23	Sumel Ashique, Biplab Debnath, Mohammad Ramzan, Tahreen Taj et al. "A critical review of the synergistic potential of targeting p53 and CAR T cell therapy in cancer treatment", Biomedicine & Pharmacotherapy, 2025<br>Publication	<1 %
24	Wenwen Wei, Dong Yang, Xi Chen, Dandan Liang, Liqun Zou, Xudong Zhao. "Chimeric antigen receptor T-cell therapy for T-ALL and AML", Frontiers in Oncology, 2022<br>Publication	<1 %

Exclude quotes	On	Exclude matches	Off
Exclude bibliography	On		
