# Supplementary material for: CAR‐DC combined with CAR‐T therapy for relapsed/refractory acute myeloid leukaemia: Research progress and future perspectives
Source: Clin Transl Med. 2025 Nov 25;15(12):e70536. doi: 10.1002/ctm2.70536 (PMC12647367; doi:10.1002/ctm2.70536)
Supplement: Supplementary file 2 — Supporting Information [file CTM2-15-e70536-s002.pdf]

5a

作者为 liriou noieqe

提交日期: 2025年11月01日 12:18下午 (UTC+0300)
提交作业代码: 2799712144
文档名称: aaaa2.docx (59.5K)
文字总数: 6910
字符总数: 44776

CAR-DC Combined with CAR-T Therapy for Relapsed/Refractory Acute Myeloid Leukemia: Research Progress and Future Perspectives

Rui Zhang[1*], Jinlin Zhang[2], Hongkai Zhang[3], Mingfeng Zhao[1#]

# Corresponding Author: Mingfeng Zhao, Department of Hematology, Tianjin First Central Hospital, No.2 Baoshanxi Rd, Xiqing District, Tianjin 300380, China; email: mingfengzhao@sina.com;

Formatted: Shadow

Abtract

Acute myeloid leukemia (AML) remains the most common type of leukemia in adults. Despite advances in conventional therapies, high relapse rates persist, underscoring the need for novel approaches such as Chimeric Antigen Receptor T (CAR-T) cell therapy. CLL1-targeted CAR-T emerges as a promising treatment for relapsed/refractory (R/R) AML. Although approximately 70% patients achieved remission, only a subset achieved minimal residual disease (MRD) negative remission, which still has much room for improvement. The main reasons for the failure of CLL1 CAR-T cell therapy include: (1) Persistence of CLL1-negative AML cells persist due to antigen escape; (2) Downregulation of IL-12 and other cytokines by the immunosuppressive tumor microenvironment (TME) , contributing to the exhaustion of both endogenous T cells and CLL1 CAR-T cells.

We synthesize a combination of CAR-engineered dendritic cells (CAR-DCs) and CLL1 CAR-T cells to overcome current limitations. CAR-DCs enhance antigen cross-presentation to activate endogenous T cells against antigen-negative clones, secrete immunostimulatory cytokines (e.g., IL-12) to sustain CAR-T activity, and remodel the TME. Key challenges involve optimizing CAR designs (e.g., incorporating Fms-like tyrosine kinase 3 ligand (FLT-3L) or CD40 signaling domains), mitigating toxicity, and establishing clinical administration protocols.

In this review, we provided a focused discussion on the specific challenges limiting CLL1-targeted CAR-T-cell therapy in R/R AML—namely, antigen escape and the TME—and argued for the novel combination strategy of CAR-DCs with CLL1 CAR-T cells as a promising solution to these challenges. Here, we critically examined the rationale, current research progress, and future perspectives of this synergistic approach.

Formatted: Shadow

Keywords: CLL1 CAR-T; CAR-DCs; Acute myeloid leukemia;

1. The current research status of AML treatment

The conventional treatment of Acute myeloid leukemia (AML) mainly includes chemotherapy, targeted therapy and hematopoietic stem cell transplantation, but most patients face the risk of relapse[1-2]. Chimeric Antigen Receptor T (CAR-T) therapy represents a promising novel approach for relapsed/refractory (R/R) AML, although significant challenges remain. Currently, CAR-T cell therapy has achieved impressive clinical outcomes in hematologic malignancies, including acute lymphoblastic leukemia, lymphoma and multiple myeloma, by targeting CD19, CD22, and BCMA[3-6]. However, CAR-T cell therapy remains clinically immature for AML, with limited overall efficacy in current studies[7-8]. The primary reasons include the lack of specific targets[9-10], the immunosuppressive tumor microenvironment (TME)[11-12], and antigen escape[13-15]. Early CAR-T therapies for AML targeted CD33 and CD123, but their toxicity or poor efficacy has limited their clinical application[7,16-17]. Other potential targets include CLL1, FLT3, NKG2D, CD7 and CD38, etc[18-23]. CLL1-targeted CAR-T cells have demonstrated promising efficacy in the treatment of R/R AML.

2. Therapeutic Efficacy of CLL1 CAR-T Cells in Relapsed/Refractory AML

CLL1 (C-type lectin-like molecule-1) is a C-type lectin-like receptor that is highly expressed on leukemia stem cells (LSCs) (about 45%) and leukemia progenitor cells (77.5-92%). Tashiro et al. developed the first CLL1 CAR-T cells, which selectively killing leukemic progenitor cells and leukemia cells[24]. CLL1 CAR-T cells have shown superior AML killing in vitro and in mice[18]. A case report described complete remission in a 10-year-old patient after CLL1 CAR-T therapy[25]. Our group reported a 70% complete remission rate in 10 adult AML patients treated with CLL1 CAR-T cells[26]. At present, a total of 48 patients received CLL1 CAR-T cell therapy. All patients underwent efficacy evaluation between days 14 and 16 post-infusion. Complete remission (CR) was achieved in 34 patients (70.83%), including 13 with minimal residual disease (MRD)-positive CR and 21 with MRD-negative CR. The

Formatted: Shadow

remaining 14 patients (29.17%) showed no response to the treatment. Regarding adverse events, cytokine release syndrome (CRS) and immune effector cell-associated neurotoxicity syndrome (ICANS) occurred in 95.83% and 20.83% of patients, respectively. Severe (grade 3/4) CRS and ICANS were observed in 41.67% and 10.42% of patients. Hematologic toxicities were the most common complications, with near-universal incidence of leukopenia, granulocytopenia, anemia, and thrombocytopenia. Notably, severe and prolonged granulocytopenia significantly increased the risk of infectious complications. Among the reported infections, 38 patients experienced bacterial infections, while viral and fungal infections were documented in 9 and 10 patients, respectively. Comparative analysis further revealed that patients with severe CRS (grade 3/4) exhibited more pronounced cytokine release, particularly elevated levels of IL-2, IL-6, IL-10, C-reactive protein (CRP), and ferritin, compared to those with mild CRS (grade 1/2)[27]. Although CLL1-targeted CAR-T cells have shown clinical efficacy in R/R AML, approximately 30% of patients exhibit primary treatment resistance. Furthermore, only 50% of responders achieve minimal residual disease (MRD)-negative remission, underscoring the need for enhanced therapeutic strategies.

The key limitations include the following: (1) Antigen escape: AML cells with low expression of CLL1 are difficult to recognize by CAR-T cells, and relapsed patients often exhibit weak expression of target antigens[28-29]. (2) TME and IL-12: The TME decreases the efficacy of CAR-T cells via myeloid-derived suppressor cells (MDSCs), regulatory T cells (Tregs) and tumor-associated macrophages (TAMs)[30-32]. Additionally, dysregulated cytokine levels (e.g., IL-12) impair CAR-T cells function. Moreover, mKRAS-specific NeoCARs with inducible IL-12 secretion and T-cell receptor (TCR) knockout demonstrate potent in vivo antitumor activity and a favorable safety profile[33]. IL-12 and IFNα2 co-expression with CAR augments the proinflammatory TME and mitigates the exhaustion of T-cells[34]. Additionally, mbIL12-engineered CAR-T cells are safe and effective in overcoming the TME [35]. IL-12 not only increases the cytotoxicity of CAR-T cells but also reprograms the TME, increasing proinflammatory $CD4^+$ T-cell infiltration, reducing regulatory T-cell (Tregs) numbers, and activating the myeloid compartment. These immunotherapy-enabling

Formatted: Shadow

effects were achieved with minimal systemic toxicity in GBM-targeted CAR-T-cell therapy[36]. Therefore, combined strategies involving CAR-T-cell engineering and TME modification are essential for enhancing the clinical effectiveness of CLL1 CAR-T-cell therapy.

3. Research progress and challenges associated with DC and CAR-DC therapy

Dendritic cells (DCs) mediate antitumor immunity by phagocytosing tumor material, processing tumor antigens, and presenting peptide-MHC complexes to activate tumor-specific T cells[37-38]. Mature DCs engage directly with T cells through co-stimulatory molecules (CD80/CD86), secrete IL-12, and activate the priming of endogenous tumor-specific T cells[39-41]. Mature DCs initiate tumor-specific CD8$^+$ T-cell immunity by migrating from tumors to lymph nodes, capturing antigens, and activating naïve T cells[42].

Conventional cDCs are broadly categorized into two functionally distinct subsets: type 1 (cDC1) and type 2 (cDC2), which exhibit distinct functional roles[43]. cDC1s prime CD8$^+$ T cells in specific regions of the draining lymph nodes (dLNs). They activate Toll-like receptors (TLRs) to secrete IL-12p70 and IFN-α, driving Th1-type immunity. Due to their ability to stimulate anti-tumor immunity, cDC1s serve as a favorable prognostic marker in cancer patients[44]. cDC2s, which are more abundant than cDC1s, express surface markers such as CD11c, CSF-1R, MHC-II, CD11b, BDCA1 and SIRPα. Human cDC2s produce various cytokines, including IL-23 and IL-10, and present antigens to CD4$^+$ helper T cells, thereby activating effector T-cell subsets such as Th2 and Th17 cells[45]. Several novel DC subsets have been identified, such as LAMP3$^+$ DCs[46-47] and AXL$^+$SIGLEC6$^+$ (AS) DCs[48]. Some studies have described DC precursors in human bone marrow and blood, revealing both shared and unique regulatory networks. Precursor-like clusters only partially aligned with these reports, suggesting underlying heterogeneity within pre-DCs that may reflect early lineage bias[49-50].

Formatted: Shadow

Single-cell and bulk RNA sequencing revealed an accumulation of immunosuppressive $CCL22^{+}$DCs, termed “exhausted DCs”, which were reversed by the herbal formula SBJDD and its component berberine. TMEM131 promotes $CCL22^{+}$DC generation via TNF signaling, a process inhibited by berberine[51]. Through lineage tracing and molecular phenotyping, DC3s were identified as a distinct DC subset derived from monocyte-DC progenitors via $Lyz2^{+}Ly6C^{+}CD11c^{-}$ precursors, a developmental pathway separate from that of DC2s originating from common DC progenitors. This finding revealed that DC3 is a DC lineage that is phenotypically related to, but developmentally distinct from, monocytes[52]. ScRNA-seq revealed significant heterogeneity and alterations in major immune cells (T, B, NK, and myeloid cells) across tissues during sepsis. A distinct cDC subcluster that displays maturation, migration, and immunoregulatory gene signatures, which is consistent with the mregDCs described in cancer was identified. This sepsis-induced mregDC subset was protein-confirmed and shown to activate naïve $CD4^{+}$T cells while promoting the differentiation of Tregs[53].

The functional state is determined by maturation status: mature DCs express high levels of MHC and co-stimulatory molecules (e.g., CD80/86), promoting effector T-cell activation, whereas immature DCs often induce Tregs and immune tolerance. Tumors frequently evade immune surveillance by disrupting the maturation of DCs, making DC-targeted strategies a promising avenue for immunotherapy. In AML patients, the frequency and function of DCs are often impaired, contributing to disease progression and therapy resistance[54]. In vivo, cDC1 can specifically perform tumor antigen cross-presentation and is essential for generating adaptive antitumor immunity. Without cDC1, tumors evade immune elimination[55]. Given their exceptional capacity for the activation and cross-presentation of T cells, cDC1 is a preferred subset of CAR-DC-based immunotherapies.

DC/tumor fusion vaccines or tumor lysates loaded on DCs can mediate the activation and persistence of antitumor T cells, and expand the clonal population of T cells that target tumor cells[56]. A phase II clinical trial (NCT03059485) reported that

Formatted: Shadow

DC/AML fusion vaccination, without maintenance therapy, achieved 73% two-year overall survival (OS) and 36% progression-free survival (PFS) in elderly AML patients[57]. Eps8-DCs enhance CD19 CAR-T-cell functions, augmenting cytokine secretion, CD107a degranulation and cytotoxic activity[58]. However, DC dysfunction contributes to tumor immune evasion and limits the efficacy of DC vaccines in elderly patients[59]. The clinical efficacy of moDC vaccines remains variable, and is limited by challenges in antigen loading, incomplete maturation, and tumor heterogeneity. The secretion of IL-12 directly affects the function of endogenous DC1[57,60-61]. Conventional DC vaccines face significant limitations, including the dysfunction of DCs (especially in the elderly), poor and variable efficacy due to challenges in antigen loading and incomplete maturation, and susceptibility to tumor heterogeneity. These limitations hightlight the need for advanced strategies such as CAR-DC technology.

The TME impairs DC function through multiple inhibitory mechanisms, including: (1) induction of the apoptosis of DCs[62-63]; (2) suppression of DC maturation and antigen presentation[64-65]; (3) promotion of tolerogenic DC phenotypes[66-67] ; (4) and downregulation of DC-recruiting chemokines to limit tumor infiltration[68-71].

Further improvements include the induction of DC reprogramming. The DC growth factor Fms-like tyrosine kinase 3 ligand (Flt3L) strengthens T-cell-mediated antitumor immunity by expanding and activating DC populations[55]. Highly activated DCs can induce $CD4^{+}$T cells to acquire cytotoxic and antitumor functions in aged mice[58]. PU.1-, BATF3- and IRF8- mediated DC reprogramming reduces exhaustion and increases memory and stem cell-like T-cell infiltration[72]. Knockout of BCL9/BCL9L in cDC1 enhances $CD8^{+}$ T-cell activation, antigen presentation, epitope expansion, and promotes antitumor activity[73-74]. Although promising strategies such as Flt3L expansion and transcriptional reprogramming (e.g., via PU.1, BATF3 and IRF8) have been developed. DC reprogramming faces significant challenges. Key limitations include the translational gap between mouse models and human patients, the complexity and safety of reliably controlling genetic or transcriptional programs in clinical settings, and the instability of the reprogrammed state within the immunosuppressive tumor microenvironment.

Formatted: Shadow

Moreover, CAR-DCs can specifically recognize tumor antigens, efficiently phagocytose CAR-targeted tumor cells and debris, and subsequently activate endogenous tumor-specific T-cell responses. Antitumor T-cell responses can directly eliminate CAR-targeted antigen-positive tumors and indirectly eliminate antigen-negative tumors (which are not directly recognized by the CAR) through cross-presentation and epitope spreading[69,75]. Activated CAR-DCs can secrete immunostimulatory cytokines (e.g., IL-12[35,69,76-77]. The intracellular signal transduction domain of CAR can be continuously activated during tumor recognition, which allows DCs to maintain homeostasis and overcome the microenvironment, leading to the acclimation of tolerant DCs.

The mechanism by which CAR-DCs counteract antigen escape and target CAR antigen-negative cells lies in the unique ability of CAR-DCs to engage in antigen cross-presentation and the subsequent induction of epitope spreading. (1) Phagocytosis of antigen-positive cells: CAR-DCs use their chimeric antigen receptor (CAR) to specifically recognize and phagocytose CAR-positive (e.g., CLL1$^+$) AML cells. (2) Cross-presentation and endogenous T-cell activation: After phagocytosis, CAR-DCs process entire tumor cell contents, not just a single CAR-targeted antigen. They then present a broad repertoire of tumor-derived peptides (from various tumor-associated antigens) via MHC-I molecules to activate endogenous, non-engineered CD8$^+$T cells. This process is known as cross-presentation. (3) Elimination of antigen-negative clones via epitope spreading: These newly activated endogenous T cells are polyclonal and can recognize multiple antigens present on the original tumor cell. Consequently, they can effectively target and eliminate AML cell clones that have escaped CAR-T-cell therapy by downregulating or losing a single target antigen (e.g., CLL1-negative cells). This phenomenon, where an immune response initiated against a single antigen expands to include other antigens from the same target, is called epitope spreading.

The process by which CAR-DCs target and eliminate CAR antigen-negative cells is as follows: CAR-mediated phagocytosis → broad antigen processing/cross-presentation → activation of endogenous T cells against multiple tumor antigens → elimination of antigen-negative clones via epitope spreading.

Formatted: Shadow

4. Validation of target specificity using gene knockout (ko) models

In vitro validation often involves the use of CRISPR-Cas9 or RNA interference to knockout the target antigen (e.g., CLL1) in AML cell lines. For instance, CLL1-KO AML cells show significantly reduced susceptibility to CLL1-targeted CAR-T cell killing, while retaining sensitivity to CAR-T cells targeting alternative antigens (e.g., CD33 or CD123)[18,24]. This confirms the antigen specificity of the CAR construct. Similarly, when CAR-DCs are co-cultured with CLL1-KO AML cells, their phagocytic capacity and subsequent T cell activation are markedly diminished, underscoring the dependence on CAR-mediated recognition[69,75].

In vivo studies further validate these findings using xenograft models established with CLL1-deficient AML cells. In such models, CLL1 CAR-T cells exhibit reduced leukemic control compared to their activity against CLL1-positive tumors, highlighting the role of antigen expression in therapeutic efficacy[18,26]. Moreover, in dual-flank tumor models—where one tumor expresses CLL1 and the other is CLL1-KO—adoptive transfer of CLL1 CAR-T cells combined with CAR-DCs results in preferential regression of the CLL1-positive tumor, with limited impact on the KO tumor unless epitope spreading occurs via CAR-DC-mediated cross-presentation[75,82]. These knockout models not only confirm the on-target mechanism of action but also help elucidate escape mechanisms. For example, residual disease in CLL1-KO models often emerges from antigen-low or antigen-negative clones, reinforcing the need for combination strategies that address heterogeneity, such as the incorporation of CAR-DCs to broaden antigen recognition.

Genetic knockout models provide indispensable evidence for the specificity and mechanism of CAR-DCs and CAR-T therapies. They validate that targeting CLL1 is both necessary and sufficient for initiating antitumor responses, while also revealing limitations that inform the design of next-generation combinatorial immunotherapies.

5.CAR-DC combined with CAR-T-cell Therapy

CAR-DC is effective against acute myeloid leukemia in preclinical studies and are being studied in humans for their ability to treat solid epithelial malignancies (NCT05631899 & NCT05631886). NCT05631899 is a pilot clinical trial evaluating the

Formatted: Shadow

safety, immune response and efficacy of an EphA2-targeting CAR-DC vaccine loaded with a KRAS mutant peptide (KRAS-EphA2-CAR-DC) in combination with immune checkpoint inhibitors (ICIs) for patients with locally advanced or metastatic solid tumors. Preclinical studies have revealed that these engineered CAR-DCs improve the cytotoxicity of co-administered CAR-T cells in solid tumor mouse models. NCT05631886 is a parallel pilot trial investigating a similar EphA2-directed CAR-DC vaccine with TP53 mutant peptide (TP53-EphA2-CAR-DCs) plus ICIs, enrolling patients with solid tumors or R/R lymphomas. Several studies have suggested that the intratumoral delivery of DCs can safely potentiate CAR-T-cell activity and improve the immunosuppressive TME. This study demonstrated that the in vitro differentiation of DCs expressing 4-1BB is enriched with the $CD141^{+}$/$Clec9A^{+}$ DC subset. Moreover, CAR-DC and CAR-T-cell interactions synergistically enhance anti-AML cytotoxicity[78]. DC-derived cytokines (e.g., IL-12 and type I IFNs) provide additional T-cell stimulation during antigen presentation, while CAR-T cells retain their intrinsic tumor-killing capacity independent of the involvement of DCs[79]. These results showed that intratumoral CAR-DC delivery creates an "immunological niche". CAR-DCs secrete IL-12 and type I IFNs reverse T-cell exhaustion and the TEM. This synergy hinges on overcoming the limitations of CAR-T-cells (poor infiltration, antigen loss) through DC-mediated antigen spreading and localized immune activation. Studies have demonstrated that 4-1BB-engineered autologous DCs improve the efficacy of anti-CD33 CAR-T-cell therapy in AML by secreting cytokines and promoting CAR-T-cell recruitment to the bone marrow niche[80]. The synergistic interaction between CAR-DCs and CAR-T cells may provide a novel strategy to increase the efficacy of the antitumor effects of cancer immunotherapy.

Mechanistic overview of CAR-DC and CAR-T-cell collaboration (Figure 1): (1) CAR-DCs phagocytose AML cells via CAR targeting, process antigens, and cross-present tumor-derived peptides via MHC-I/II to activate endogenous T cells[40-41]. (2) CAR-DCs secrete cytokines (e.g., IL-12) to support the activation, proliferation, and persistence of CAR-T cells, potentially reducing exhaustion and promoting a memory phenotype[40,35,79,81]. (3) Activated endogenous T cells (primed by CAR-DCs) and CAR-T cells work together to eliminate both CLL1-positive and CLL1-negative

Formatted: Shadow

(via epitope spreading) AML clones[82-83]. The comparative characteristics of CAR-DCs are summarized in Table 1.

Therefore, CAR-DCs and CAR-T-cell therapy have good complementarity, and can enhance the function of CAR-T cells and overcome immune escape to prevent tumor recurrence. The limitations of the CAR-DC and CAR-T-cell combination strategy can be summarized as follows: (1). Early-stage clinical evidence: The efficacy of the combination is supported primarily by preclinical studies. Clinical data on humans are still nascent, and are being investigated only in early-phase pilot trials (e.g., NCT05631899 and NCT05631886), and their definitive value remains unproven. (2). Logistical and Manufacturing Complexity: The therapy involves the production, quality control, and administration of two distinct, sophisticated cellular products (CAR-DCs and CAR-T cells). This dual-product approach significantly increases the complexity, cost, and challenges of scalable manufacturing compared to single-agent therapies. (3). Unvalidated Synergy in Human Tumors: While the proposed mechanism of creating an "immunological niche" is compelling in mouse models, the efficiency and reliability of this synergy—including consistent reversal of T-cell exhaustion and effective overcoming of the immunosuppressive tumor microenvironment—require robust validation in human patients. (4). Safety Profile Uncertainties: The combined approach could potentially alter or amplify toxicity profiles, such as the risk of exacerbated cytokine release syndrome (CRS), due to the potent, reciprocal activation of two engineered immune cell types. The safety of this interaction is not yet fully characterized.

6. Design and function optimization of CAR-DCs

The selection and design of the intracellular signal transduction region of CAR is the key in DC cell therapy. It can promote DC differentiation, phagocytosis and/or antigen cross-presentation after tumor antigen recognition by CAR (Figure 2):

(1) 4-1BB signaling domain: CD33 CAR-DCs with a 4-1BB-CD3ζ intracellular domain can activate CAR-T cells and increase their antitumor efficacy in AML when co-cultured in vitro[80].

(2) Flt3L signaling domain: The University of Washington engineered CAR-DCs cells with an FLT3-integrated intracellular domain, enabling the differentiation of cDC

Formatted: Shadow

and enhancing the cross-presentation of tumor antigens[72]. Flt3L-secreting engineered T cells synergize with pattern recognition receptor (poly(I:C)) and co-stimulatory (anti-4-1BB) agonists to increase intratumoral DC accumulation and systemic antitumor immunity. This approach mitigates antigen-negative tumor escape in solid tumors[39, 75].

(3) CD40 signaling domain: The CD40 signaling cascade upregulates MHC class II, co-stimulatory molecules (CD86/CD70/CD80), and cytokines (IL-12, TNF-α), thereby potentiating antigen cross-presentation[84]. Preclinical studies have demonstrated that CAR-DCs with a CD40 intracellular domain, when combined with BCMA CAR-T cells, increase the efficacy of multiple myeloma treatment[23].

Therefore, selecting the appropriate intracellular domain (e.g., 4-1BB, FLT3, or CD40) of CAR can induce the mature phenotype of CAR-DCs when recognizing the tumor, and overcome the acclimation of tolerant DCs by the suppressive tumor microenvironment.

7. Emerging technologies and future directions:

(1) iPSC-Derived Allogeneic CAR Products: iPSC-derived allogeneic CAR products represent a promising off-the-shelf approach to overcome the high cost, complex manufacturing, and batch variability associated with autologous cell therapy[85]. FT819 (anti-CD19 CAR-T cells with a TRAC-integrated CAR) showed no dose-limiting toxicity, GvHD, or severe CRS in an ongoing phase I trial, with four out of 15 patients responding. FT825/ONO-8250 (anti-HER2, seven edits) aims to enhance solid tumor targeting. Their iCAR-NK products, including FT596 (anti-CD19 + hnCD16 + IL-15RF), which induced responses in nine out of 17 patients, and FT522/FT576 (with added edits for durability and combination therapy), also revealed favorable safety and efficacy. Century Therapeutics CNTY-101, a multi-edited CD19-targeting iCAR-NK product, showed no significant toxicity and a 40% response rate in a phase I trial. These advances highlight the potential of iPSC-derived CAR cells to provide standardized, scalable and safer allogeneic immunotherapies[86]. The potential of iPSCs lies in their ability to create unlimited, standardized, "off-the-shelf" CAR-DCs and CAR-T cells—thereby addressing the cost, complexity,

Formatted: Shadow

and batch variability that plague autologous dual-cell therapies.

The generation of functionally mature iPSC-derived CAR-T (iCAR-T) cells faces several technical and biological hurdles. The available differentiation protocols, yield innate-like T cells (iT cells) that express CD8αα and CD56 but lack CD8αβ, CD2, CD5, and CD28[87-90]. A key challenge is impaired transition through the double-positive (DP) stage, which is essential for conventional αβ T-cell development.

Premature TCR or CAR expression, which often occurs when T-cell-derived iPSCs or constitutive CAR constructs, are used, inhibits DP formation and skews differentiation toward innate $CD8\alpha\alpha^+$ or γδ T-like lineages. This is mediated through the suppression of Notch signaling (reduced Notch1/3 and downstream targets) and downregulation of PTCRA, a gene required for the development of αβ T-cells [89,91] .

Strategies to improve T-lineage differentiation include the following: removing double-negative T cells to prevent the killing of DP progenitors; using OP9-DLL4 or 3D thymic cultures to increase Notch signaling; optimizing the CAR design: incorporating 4-1BB (rather than CD28) co-stimulatory domains to improve DP transition and $CD8\alpha\beta^+$ output; targeting TRAC insertion of the CAR with attenuated ITAMs to prevent tonic signaling and preserve Notch/PTCRA expression; and providing the CAR antigen and 4-1BBL during differentiation to promote DP-to-SP progression. Most protocols predominantly generate $CD8^+$ iCAR-T cells, even when starting from $CD4^+$ T cells. Co-culture with artificial thymic organoids enables the production of $CD4^+$ iT cell , and a recent feeder-free approach using PMA/ionomycin to skew DP cells toward the $CD4^+$ lineage offers a promising scalable alternative. Achieving balanced $CD4^+$/$CD8^+$ compositions may increase the efficacy of antitumor effects and persistence of iCAR-T-cell products. This highlightes the challenge of dual genetic engineering (e.g., introducing a CAR while maintaining DC differentiation potential in iPSCs) and the need to eliminate alloreactivity through strategies such as HLA editing, which adds another layer of complexity.

(2) In vivo CAR Cell programming: Direct in vivo CAR gene delivery can intrinsically activate T cells, for example, via innate immune sensing triggered by

Formatted: Shadow

RNA in LNPs. Unlike strong artificial stimulation during ex vivo manufacturing, in vivo generated CAR-T cells expand gradually under physiological antigen drive, preserving stem-like properties, avoiding exhaustion, and enabling sustained antitumor activity[92]. In vivo delivery may selectively target less differentiated T cells, such as naive (Tn), stem-like memory (TSCM) or central memory (TCM) subsets, which possess greater expansion, persistence and antitumor potency. Reprogramming even a few of these cells can induce robust and durable responses. Local administration (e.g., intratumoral) can further enhance this effect by engineering tissue-resident memory T cells (TRM) cells, enabling direct tumor targeting without hindering migration[93]. Dynamic TME activation: In vivo-generated CAR-T cells undergo progressive activation within the tumor microenvironment. Synergy with the host immune system: Since in vivo CAR-T cells often require no or mild lymphodepletion, the host immune system remains largely intact[94].

The available ex vivo CAR-T-cell therapeutic strategies require complex, week-long manufacturing processes, and are limited by patient-specific T-cell quality. In vivo CAR-T cells directly deliver CAR-encoding genetic vectors (viral or non-viral nanoparticles) to the patients.

Two major systems are used: viral vectors (e.g., AAV and lentivirus), which offer long-term expression without integration but lack natural T-cell tropism. Lentiviruses can be pseudotyped with targeting ligands (e.g., anti-CD3/CD8 scFv) to increase the specificity of T-cells. Studies have shown successful in vivo CAR-T generation for the clearance of $CD19^+$ B malignant cells[95-96]. Challenges: Significant safety concerns, including insertional mutagenesis, off-target transduction (e.g., germline or malignant cells), and pre-existing immunity. Non-viral platform: Nanoparticles vectors: Lipid nanoparticles (LNPs) or polymer-based nanoparticles. Payload: Primarily CAR-encoding mRNA (for transient expression) or DNA (for potential stable integration, e.g., using the piggyBac transposon system). Non-viral nanoparticles (e.g., polymers and LNPs): Polymer/LNP nanoparticles encapsulate CAR mRNA/DNA for transient/stable expression. Untargeted LNPs mainly accumulate in the liver/spleen; targeted LNPs decorated with T-cell-specific

Formatted: Shadow

antibodies (e.g., anti-CD5) improve the delivery. The polymer nanoparticles delivering CAR mRNA against prostate/liver cancer antigens inhibited tumor growth in mice[97]. AAV-based in vivo CAR-T generation led to tumor regression in mouse models[98]. Loop33 and 123 CAR-T cells targeting CD33 and CD123 efficiently eliminate AML cells and prolong the survival of tumor-bearing mice, while addressing the issue of immune escape[99]. Challenges: Transient expression requires multiple doses, antibody conjugation complicates manufacturing and potential immunogenicity. Limitations: (1). Delivery efficiency and specificity: The main challenge lies in achieving efficient and specific gene delivery exclusively to the desired T-cell subsets in vivo, while avoiding off-target transduction. (2).Safety control: Unlike ex vivo processes, CAR-T cells generated in vivo lack direct quality control. There is no option for phenotypic validation or the use of safety switches (e.g., suicide genes) before administration. (3). Transient expression risk: Non-viral methods (e.g., mRNA/LNPs) may lead to only transient CAR expression, potentially limiting the duration of antitumor activity and necessitating the administration of repeated doses.

Logic-gated and synthetic gene circuits: Boolean logic gates (e.g., "OR", "AND", and "NOT") are recent developments in the use of CAR-T cells as safety switches.

The simplest implementation of AND gates involves targeting two TAAs co-expressed on cancer cells but not normal tissues, inducing cytotoxicity only upon dual antigen engagement[100]. For T-cell engagers (TCEs), the selective killing of dual-TAA$^+$ cells arises from optimized cooperative binding (pseudo-avidity)[101]. Similarly, a trispecific TCE against B7-H4 and LY6E shows preferential activity in colorectal cancer[102]. The recently developed ISB2001 (anti-BCMA/CD38) acts as a strict AND-gate, with activity decreasing 100-fold after single-target deletion[103]. Split systems offer an alternative strategy. Hemibody TCEs incorporate isolated VH/VL anti-CD3 domains on separate molecules, which reassemble only on dual-TAA$^+$ cells[104]. The precision GATE platform uses half-life-extended hemibodies and protease masks to improve functionality[105-106]. Simultaneously, split CARs

Formatted: Shadow

separate activation and costimulation signals. For example, a low-affinity CD138 (stimulatory) CAR combined with a high-affinity CD38 (co-stimulatory) CAR effectively distinguished myeloma tissue from healthy tissue[107]. Tousley et al. developed a leak-resistant AND-gate CAR by incorporating LAT and SLP76 cytosolic domains, which require dual antigen engagement for activation[108]. Moreover, IF-THEN logic gates enable tumor-dependent transgene expression. SynNotch CARs, such as an anti-EGFRvIII receptor driving expression of an EphA2/IL13Rα2-targeting CAR, reduce antigen escape in glioblastoma[109] and are now in clinical testing (NCT06186401).

OR gate designs incorporate two or more binders into a single CAR [110-112] or use two or more mono-specific CARs within the same vector[113-116]. Bicistronic CARs further offer the flexibility to incorporate distinct co-stimulatory domains on each CAR, thereby broadening the spectrum of T-cell stimulation signals[117-118]. Target selection is necessary to ensure that binding to one antigen does not interfere with binding to the other—a consideration especially important for tandem CARs [111]. Dual or triple targeting has been extensively investigated in B-cell lymphoma using CAR-T-cell therapy[119-120], where antigen loss acts as a mechanism of relapse[121]. Since B-cell aplasia is generally clinically manageable, simultaneously targeting multiple B-cell lineage markers, such as CD19, CD20, and CD22, has become a common OR gate strategy[122-123]. For example, Tian et al. developed a bicistronic CAR construct targeting GPC2 and B7-H3 for neuroblastoma[124]. Although both targets are overexpressed in neuroblastoma relative to normal tissues, their expression is highly heterogeneous within tumors. By conducting CITE-Seq profiling, the authors identified CARs against each target that promoted optimal T-cell expansion and phenotype. Compared to mono-CAR-T cells, the resulting bicistronic CAR-T cells effectively killed both single- and DP tumor cells in vitro and in vivo, and demonstrated improved persistence and reduced exhaustion in mixed xenograft models. Another innovative approach involves engineering CAR-T cells to secrete bispecific TCEs that target a second antigen, effectively creating a localized OR gate system[125].

Formatted: Shadow

NOT gate logic relies on differential expression of a protective antigen present in normal cells but not tumor cells—analogous to the inhibition of NK cells via KIR receptors[126]. Inhibitory CARs (iCARs) contain ITIM domains that suppress T-cell activation upon binding to such an antigen. Early iCARs using PD-1 or CTLA-4 intracellular domains offered reversible and rapid inhibition, outperforming irreversible kill switches[127-128]. inhancing iCAR avidity and incorporating dual-inhibitory domains (e.g., PD-1 combined with LAIR-1 or SIGLEC-9) significantly reduces this delay and improves the precision of Boolean logic-gated CAR T cells[129]. The key design parameters include the iCAR avidity and expression level relative to the activating CAR. While increasing iCAR affinity alone may not improve inhibition, enhancing avidity, for example, through increased expression or antigen density, can significantly increase the efficiency of suppression[130]. Notably, iCARs may more effectively block proliferation and cytokine production than immediate cytotoxicity, which relies on pre-formed granules[131]. A clinically promising approach uses HLA loss of heterozygosity (LOH), a common event in tumors. The Tmod platform uses an inhibitory CAR (blocker) against HLA-A*02 (frequently lost in tumors) paired with an activating CAR against a tumor antigen such as MSLN or CEA[132-133]. A TAA and β2-microglobulin (β2M) with high affinity bind to CD3 with low affinity. This may inhibit the activation of normal cells (TAA$^+$/β2M$^+$) but allow the killing of tumor cells that lose β2M[134]. Our team has engineered innovative CD16-CLL1 iCAR-T cells, which preserve cytotoxicity against tumor cells while preventing the elimination of neutrophils, thereby signifficantly reducing the incidence of granulocytopenia during CAR-T-cell therapy[135]. Limitations: (1). Design complexity and off-target risks: The molecular engineering of multi-antigen systems (e.g., tandem CARs, split signals) is intrinsically complex. This can lead to unforeseen functional issues, such as scFv interference in OR-gate constructs or "leaky" activation in AND-gate circuits, potentially resulting in on-target, off-tumor toxicity. (2). Stringent dependency on the tumor antigen profile: The efficacy of these strategies greatly depends on ideal antigen expression patterns, which are often limiting. AND gates require tumor-specific co-expression of two

Formatted: Shadow

antigens, whereas NOT gates rely on a "protective" antigen being consistently absent on tumors. Tumor heterogeneity and antigen loss are potent escape mechanisms. (3). Kinetic and potency challenges: There are fundamental biological hurdles. For example, inhibitory signals from iCARs (NOT gates) may be too slow to prevent immediate cytotoxicity to healthy tissues. Moreover, complex signaling architectures can sometimes weaken overall T-cell activation and decrease antitumor potency compared to conventional CARs. (4). Unproven clinical translation: Most logic-gated approaches are in preclinical or early-phase clinical trials. Their greater efficacy and safety profiles in humans have not yet been definitively established. The immunosuppressive TME may also disrupt the delicate logic of these engineered circuits.

Armored CAR designs: Several strategies to improve the antitumor effect of adoptive T-cell therapy involve the expression of a cytokine transgene in CAR-T cells. The use of CAR-T cells, which provide autocrine growth factors and stimulatory cytokines is one approach to protect or "armor" the T cells from the suppressive tumor environment. IL-2 transgene: IL-2 supports T-cell growth but causes significant toxicity at therapeutic doses. Early studies have shown that T cells engineered to secrete IL-2 proliferate without exogenous cytokine support and mediate tumor regression in melanoma models[136-138]. However, clinical trials revealed toxicity without improved efficacy[139], limiting its use. IL-12 transgene: IL-12 is a potent immunostimulatory cytokine but is highly toxic when delivered systemically[140]. Localized expression within tumors via CAR T cells may reduce systemic exposure. In murine models, IL-12-secreting pmel T cells increase antitumor activity and remodel the tumor microenvironment by modulating myeloid cells[141-143] Inducible expression systems (e.g., NFAT promoters) improve safety while maintaining efficacy[144-145]. Studies using CD19-targeted CAR/IL-12 T cells have demonstrated complete tumor eradication and resistance to Treg suppression in syngeneic models[146]. IL-15 transgene: IL-15 promotes the survival and proliferation of T cells and NK cells[147-149]. Constitutive IL-15 expression was shown to enhance CAR-T-cell persistence and antitumor activity in preclinical studies[150-151]. However, uncontrolled

Formatted: Shadow

proliferation and leukemogenic risks were observed in human T cells[152]. Co-expression of suicide genes (e.g., iCaspase9) improved safety, enabling rapid elimination of over-proliferating cells[153]. IL-21 culture and transgene: Ex vivo culture with IL-21 enhanced CAR-T-cell expansion, cytotoxicity, and in vivo efficacy against Nalm6 tumors[154]. Whether IL-21 transgene expression can safely provide autocrine stimulation is under investigation. Limitations of the armored CARs: (1). Cytokine-related toxicity: The constitutive or induced secretion of potent immunostimulatory cytokines (e.g., IL-12 and IL-15) carries a significant risk of severe systemic toxicity, including CRS and neurotoxicity (ICANS), even with localized expression strategies. (2). Challenges in controllability: Precisely regulating the timing, level, and duration of cytokine production is difficult. Constitutive expression, particularly of cytokines such as IL-15, poses a risk of uncontrolled T-cell proliferation and potential leukemogenic transformation, necessitating complex safety switches such as suicide genes. (3). Narrow therapeutic window: Clinical evidence (e.g., with IL-2) suggests that augmenting CAR-T cells with cytokines may not always improve efficacy in proportion to the increase in toxicity, resulting in a narrow therapeutic index. (4). Immature clinical validation: The long-term safety and efficacy of most advanced CAR designs, especially those using inducible systems or cytokines such us IL-21, remain unproven in large-scale clinical trials.

Multiple cell types for UCAR-T-cell generation: Several immune cell types are ideal sources for allogeneic UCAR-T-cell manufacturing because of their low risk of graft-versus-host disease (GvHD). These include γδ T cells, invariant natural killer T cells (iNKTs), double-negative T cells (DNTs), and virus-specific T cells (VSTs), as well as engineered sources such as induced pluripotent stem cells (iPSCs) and placental circulating T (P–T) cells. Their unique antigen recognition mechanisms may also improve efficacy against solid tumors.

γδ T cells: γδ T cells (~5% of peripheral $CD3^+$ cells, mainly the Vγ9Vδ2 subtype) recognize antigens in an HLA-independent manner, minimizing the risk of GvHD[155]. Their intrinsic antitumor activity allows them to be targeted even after CAR antigen loss, addressing antigen heterogeneity[104]. Adicet Bio's CD20-targeted γδ UCAR-T

Formatted: Shadow

(ADI-001) showed a 67% ORR and CR in B-cell malignancies, with dose-dependent expansion despite HLA mismatch[156-157]. Their dual targeting via native TCR and NKG2D increases their efficacy, for example, temozolomide upregulates NKG2DL in glioblastoma, increasing the sensitivity of γδ T cell [158].

iNKT cells: iNKT cells recognize glycolipids via CD1d, an HLA-independent mechanism that reduces GvHD risk[159]. They home to tumors via chemokines (e.g., CCL2/CCL20), making them suitable for solid tumors[160]. CAR-iNKTs have been studied in hematologic and solid malignancies[161-162]. A clinically advanced allogeneic CD19-CAR iNKT product co-expressing IL-15 and shRNAs against B2M/CD74 (to reduce HLA-I/II) showed efficacy in patients with relapsed/refractory NHL and ALL[163]. Autologous GD2-CAR iNKTs with IL-15 induced a CR in neuroblastoma[164]. Besides direct cytotoxicity, CAR-iNKTs engage in host immunity by cross-priming $CD8^+$ T cells[107], depleting immunosuppressive $CD1d^+$ TAMs and MDSCs[165-166], activating DCs, and promoting epitope spreading[108].

DNTs: DNTs ($CD3^+CD4^-CD8^-$) rarely cause GvHD. Allogeneic DNTs expanded from AML patients showed safety and antitumor activity via NKG2D/DNAM-1[109,167-170]. CD19-CAR-DNTs effectively targeted B-cell leukemia and lung cancer without GvHD[171]. CAR4-DNTs against T-cell malignancies showed greater persistence with idelalisib[172]. A phase I trial of allogeneic CD19-CAR-DNTs (RJMty19) in B-cell lymphoma reported no ≥G3 CRS, ICANS, GvHD, or DLTs; all high-dose patients responded[173].

VSTs and engineered cell sources: VSTs have a restricted TCR repertoire, lowering the risk of GvHD [174-175]. They are used against viral infections post-HSCT[176], but their anti-cancer application remains limited[177-178].

iPSCs offer a renewable CAR-T source: CAR-iPSCs differentiated via 3D organoids yield functional T cells with uniform TCRs and low MHC expression, reducing GvHD/rejection risks[179]. Inhibiting G9a/GLP enhances the maturation of iPSC-T-cells and CAR effector function[180].

Limitations of allogeneic UCAR-T-cell sources: （1）. Cell source scarcity and expansion difficulties: Key candidate cells, such as γδ T cells, iNKT cells, and DNTs

Formatted: Shadow

are rare in peripheral blood, making their isolation, genetic engineering, and large-scale expansion challenging and costly. (2). Uncertain persistence and potency: Some cell types, such as VSTs, may have limited in vivo persistence and expansion capacity, potentially restricting their long-term antitumor activity. (3). Host versus-graft rejection (allo-rejection): The host immune system can recognize and eliminate allogeneic CAR-T cells due to HLA mismatching, leading to rapid rejection and short persistence. While strategies such as HLA editing (in iPSCs) can mitigate this, they cannot completely eliminate the risk. (4). High technical complexity: The manufacturing process, especially for iPSC-derived CAR-T cells, is highly complex and involves reprogramming, gene editing, and differentiation, presenting significant hurdles in terms of quality control, standardization, and scalability. (5). Unconventional safety profiles: The native reactivity of γδ T cells or iNKT cells can lead to on-target, off-tumor toxicity in healthy tissues, which requires careful evaluation.

4. Current challenges and limitations related to CAR-DCs

The plasticity of DCs and intrinsic dysfunction in AML: AML intrinsically subverts the differentiation of DCs, as indicated by the predominance of arrested DC precursors (Lin$^-$HLA-DR$^+$CD11c$^+$CD123$^+$) and deficiency in terminal DC subsets (BDCA-1$^+$/BDCA-3$^+$mDCs; BDCA-2$^+$pDCs) in FLT3-ITD$^+$patients at diagnosis. The impairment of myeloid DCs persists even in remission[181], suggesting inherent defects in the maturation of DCs that may compromise the functionality of CAR-DCs. Moreover, conventional chemotherapeutic agents (e.g., daunorubicin) exacerbate immunosuppression by inducing ATP release from dying blasts, activating the P2X7-IDO1 axis in DCs to drive Treg expansion and tolerogenesis[182]. This raises concerns that AML-educated DCs and CAR-DCs may retain aberrant plasticity or immunosuppressive traits.

Tumor-induced tolerogenic DC phenotypes: (1) Metabolite-driven tolerance: The mregDCs exhibit a migratory capacity to tumor-draining lymph nodes, where they mediate the trans-suppression of DC-mediated antigen cross-presentation. The

Formatted: Shadow

mregDCs promote the differentiation of T helper 2 (Th2) cells and Tregs. Tumor-secreted lactate induces the activation of sterol regulatory element-binding protein 2 (SREBP2) in DCs, triggering mevalonate pathway-dependent differentiation into mregDCs, which suppress $CD8^{+}$T cells and promote Th2/Treg responses[64]. (2) Stromal signaling: CAF-secreted WNT2 inhibits the differentiation of DCs via SOCS3/p-JAK2/p-STAT3 signaling, blunting antitumor immunity[65]. These pathways highlight a key limitation: infused CAR-DCs may undergo "re-education" by the TME, adopting tolerogenic phenotypes (e.g., $IDO1^{+}CD39^{+}$DCs or mregDCs) that undermine CAR-T-cell collaboration. (5). Logistical and biological complexity: Manufacturing challenges: Dual-cell products (CAR-DCs+CAR-T) increase cost/complexity compared to single-agent therapeuic strategies. Lack of predictive biomarkers: It is unclear which patients benefit most from synergy (e.g., TP53/KRAS mutational status alone may be insufficient).

Preclinical evidence suggests that CAR-DCs have several theoretical advantages: (1). Precision in antigen source: Conventional DC vaccines rely on undefined tumor lysates or a limited set of known peptides/antigens. In contrast, CAR-DCs actively and specifically search for tumor cells in vivo, ensuring that they acquire an authentic and diverse set of tumor antigens directly from TME of the patients. This approach is valuable for heterogeneous tumors such as AML. (2). Overcoming the dysfunction of DCs: In AML, the endogenous DC pool is often dysfunctional. CAR-DCs are engineered to have a mature, activated phenotype upon CAR signaling, which may help them resist the immunosuppressive TME better than conventionally matured DCs. The incorporation of signaling domains like CD40 or 4-1BB can specifically designed to enhance their maturation and immunostimulatory capacity persistently. (3). Synergistic positioning: The primary rationale for CAR-DCs in our proposed combination strategy is not only to be a better vaccine, but also to act as an in situ immune orchestrator that directly supports concurrently administered CAR-T cells by secreting cytokines (e.g., IL-12) and promoting epitope spreading, functions not inherently optimized in conventional DC vaccines. CAR-DCs combined with CLL1 CAR-T cells constitute a new strategy to overcome the bottleneck of the existing efficacy for R/R AML. An

Formatted: Shadow

in-depth understanding of the mechanism of action of CAR-DCs may contribute to the clinical translation of CAR-DCs and provide a scientific basis for the combination of CAR-DCs and CLL1 CAR-T cells in the treatment of refractory or relapsed AML patients.

CAR-DCs present several challenges: 1) Functional complexity: The efficacy of the indirect "bystander" killing of antigen-negative tumors relies entirely on successful cross-presentation and epitope spreading, processes that may be inefficient in immunosuppressive environments. 2) Safety concerns: While the synergistic potential of CAR-DCs and CAR-T cells offers a promising strategy to enhance antitumor efficacy, it is crucial to address the potential safety implications of this combination. Notably, both cell types are engineered for potent immune activation, which raises concerns about exacerbated immune-related adverse events, particularly CRS. Preclinical models of CAR-DCs and CAR-T co-administration have shown enhanced cytokine production (e.g., IL-6, IL-12, IFN-γ), which could theoretically amplify CRS severity. However, existing evidence also suggests that CAR-DCs may promote a more controlled immune activation through localized cytokine secretion and epitope spreading, potentially mitigating systemic toxicity compared to conventional CAR-T monotherapy. To date, clinical trials evaluating CAR-DCs (e.g., NCT05631899) have not reported severe CRS in early-phase studies, though patient numbers remain limited. Future clinical protocols should incorporate stringent monitoring, prophylactic management strategies (e.g., tocilizumab, corticosteroids), and possibly inducible safety switches (e.g., caspase-based suicide genes) to enable rapid intervention if uncontrolled activation occurs. Therefore, while the combination holds significant therapeutic promise, its safety profile must be rigorously evaluated in phased clinical trials to balance efficacy and toxicity. 3) TME resistance: While engineered to secrete cytokines such as IL-12, the durability of this function and the ability of CAR-DCs to resist reprogramming into a tolerogenic state by the TME remain unproven. 4) CAR design: The FLT3/CD40 signaling domains need to be

Formatted: Shadow

prioritized. Future studies should define patient selection criteria and integrate biomarkers (e.g., TME profiling).

Figure lends:

Figure1: Mechanistic overview of CAR-DC cells and CAR-T cell collaboration: (1) CAR-DC cells phagocytoses AML cells via CAR targeting, processes antigens, and cross-presents tumor-derived peptides via MHC-I/II to activate endogenous T cells. (2) CAR-DC secretes cytokines (e.g., IL-12) to support the activation, proliferation, and persistence of CLL1 CAR-T cells, potentially reducing exhaustion and promoting a memory phenotype. (3) Activated endogenous T cells (primed by CAR-DC) and CAR-T cells work together to eliminate both CLL1-positive and CLL1-negative (via epitope spreading) AML clones.

Figure2: The optimal intracellular signal of CAR-DC : Selecting the appropriate intracellular domain (CD3ζ ; CD3ζ+4-1BB ; FLT3L ; CD40 and CD40+FLT3L) of CAR can induce the mature phenotype of CAR-DC.

Formatted: Shadow

5a

原创性报告

13%	9%	11%	1%
相似指数	网际网络来源	出版物	学生文稿

主要来源

1	www.ncbi.nlm.nih.gov<br>网际网络来源	3%
2	pmc.ncbi.nlm.nih.gov<br>网际网络来源	1%
3	Olivier Nolan-Stevaux, Richard Smith. "Logic-gated and contextual control of immunotherapy for solid tumors: contrasting multi-specific T cell engagers and CAR-T cell therapies", Frontiers in Immunology, 2024<br>出版物	1%
4	docksci.com<br>网际网络来源	1%
5	Tianyi Ma, Xiaojing Chu, Jinyu Wang, Xiangjie Li et al. "Pan-cancer Analyses Refine the Single-Cell Portrait of Tumor-Infiltrating Dendritic Cells", Cancer Research, 2025<br>出版物	<1%
6	Xiaoyuan He, Xiaomei Zhang, Zhengyu Yu, Juanxia Meng, Yanyu Jiang, Yifan Zhao, Hairong Lyu, Xue Bai, Xia Xiao, Mingfeng	<1%

Zhao. "Anti-FcRL5 CAR-T exhibits anti-MM activity against EMM after progression of anti-BCMA and anti-GPRC5D CAR-T: a case report", Blood Advances, 2025
出版物

---

7 translational-medicine.biomedcentral.com <1%
网际网络来源

---

8 Luca Legato, Matteo Bisio, Filippo Fasano, Corrado Benevolo Savelli et al. "Mechanisms of Resistance to CAR T-Cells and How to Overcome Them", Methods and Protocols, 2025 <1%
出版物

---

9 Castelli, Sofia. "Reprogramming CAR T Cells Through Ectopic IL-9 Signaling to Enhance Efficacy Against Solid Tumors.", University of Pennsylvania <1%
出版物

---

10 Talha Badar, Alak Manna, Martha E. Gadd, Mohamed A. Kharfan-Dabaja, Hong Qin. "Prospect of CAR T-cell therapy in acute myeloid leukemia", Expert Opinion on Investigational Drugs, 2022 <1%
出版物

---

11 Submitted to University of Birmingham <1%
学生文稿

---

patents.google.com

12	网际网络来源	<1%
13	www.1stoncology.com<br>网际网络来源	<1%
14	www.science.gov<br>网际网络来源	<1%
15	classic.clinicaltrials.gov<br>网际网络来源	<1%
16	Thuy Anh Bui, Haoqi Mei, Rui Sang, David Gallego Ortega, Wei Deng. "Advancements and challenges in developing invivo CAR T cell therapies for cancer treatment", eBioMedicine, 2024<br>出版物	<1%
17	www.frontiersin.org<br>网际网络来源	<1%
18	Andy Kah Ping Tay. "CAR-T Manufacturing - Technologies and Innovations", CRC Press, 2025<br>出版物	<1%
19	Hui Zhang, Wen-Ting Gan, Wen-Ge Hao, Peng-Fei Wang, Zhuo-Yan Li, Lung-Ji Chang. "Successful Anti-CLL1 CAR T-Cell Therapy in Secondary Acute Myeloid Leukemia", Frontiers in Oncology, 2020<br>出版物	<1%

20 Ning Jiang, Zhaoyi Yang, Huilei Miao, Shujun Xing, Shuhang Wang, Ning Li. "Recent advances in universal chimeric antigen receptor T cell therapy", Journal of Hematology & Oncology, 2025 <1 %

出版物

21 Chunrun Qu, Hao Zhang, Hui Cao, Lanhua Tang et al. "Tumor buster - where will the CAR-T cell therapy 'missile' go?", Molecular Cancer, 2022 <1 %

出版物

22 Danni Xie, Xin Jin, Rui Sun, Meng Zhang, Wenyi Lu, Xinping Cao, Ruiting Guo, Yi Zhang, Mingfeng Zhao. "Bicistronic CAR-T cells targeting CD123 and CLL1 for AML to reduce the risk of antigen escape", Translational Oncology, 2023 <1 %

出版物

23 Hui Zhang, Chaoke Bu, Zhiyong Peng, Guangchao Li et al. "Characteristics of anti-CLL1 based CAR-T therapy for children with relapsed or refractory acute myeloid leukemia: the multi-center efficacy and safety interim analysis", Leukemia, 2022 <1 %

出版物

24 Ruslan Medzhitov. "TOLL-LIKE RECEPTORS AND INNATE IMMUNITY", Nature Reviews <1 %

Immunology, 11/2001
出版物

25	Submitted to University of Texas Health Science Center<br>学生文稿	<1%
26	www.onderzoekmetmensen.nl<br>网际网络来源	<1%
27	Ilaria M. Michelozzi, Efstratios Kirtsios, Alice Giustacchini. "Driving CAR T Stem Cell Targeting in Acute Myeloid Leukemia: The Roads to Success", Cancers, 2021<br>出版物	<1%
28	www.nature.com<br>网际网络来源	<1%
29	Evan W. Weber, Marcela V. Maus, Crystal L. Mackall. "The Emerging Landscape of Immune Cell Therapies", Cell, 2020<br>出版物	<1%
30	www.centerwatch.com<br>网际网络来源	<1%
31	Hui Zhang, Pengfei Wang, Zhuoyan Li, Yingyi He, Wenting Gan, Hua Jiang. "Anti-CLL1 Chimeric Antigen Receptor T-Cell Therapy in Children with Relapsed/Refractory Acute Myeloid Leukemia", Clinical Cancer Research, 2021<br>出版物	<1%

32	Somayeh Vandghanooni, Morteza Eskandani, Zohreh Sanaat, Yadollah Omidi. "Recent advances in the production, reprogramming, and application of CAR-T cells for treating hematological malignancies", Life Sciences, 2022<br>出版物	<1 %
33	assets-eu.researchsquare.com<br>网际网络来源	<1 %
34	ivy.fm<br>网际网络来源	<1 %
35	jbmtct.emnuvens.com.br<br>网际网络来源	<1 %
36	jhoonline.biomedcentral.com<br>网际网络来源	<1 %
37	www.mypublichealth.pitt.edu<br>网际网络来源	<1 %
38	www.researchgate.net<br>网际网络来源	<1 %
39	Alrefai, Hasan. "Moving Beyond M1/M2: Xenoline-Polarized Macrophages as a Physiologically Relevant Model of Tumor-Associated Macrophages in Glioblastoma.", The University of Alabama at Birmingham<br>出版物	<1 %

40 Jacob S. Appelbaum, Navin Pinto, Rimas J. Orentas. "Promising Chimeric Antigen Receptors for Non-B-Cell Hematological Malignancies, Pediatric Solid Tumors, and Carcinomas", Elsevier BV, 2020
出版物
<1 %

41 Ma, Lie. "Human Induced Pluripotent Stem Cell Derived Myeloid Derived Suppressor Cells in GVHD Therapy.", University of Minnesota
出版物
<1 %

42 S. Basta. "The Cross-priming Pathway: A Portrait of an Intricate Immune System", Scandinavian Journal of Immunology, 4/2007
出版物
<1 %

43 Wenwen Wei, Dong Yang, Xi Chen, Dandan Liang, Liqun Zou, Xudong Zhao. "Chimeric antigen receptor T-cell therapy for T-ALL and AML", Frontiers in Oncology, 2022
出版物
<1 %

44 Yongxian Hu, Jingjing Feng, Tianning Gu, Linqin Wang et al. "CAR T-cell therapies in China: rapid evolution and a bright future", The Lancet Haematology, 2022
出版物
<1 %

45 Zuyuan Luo, Zhen Liu, Zhen Liang, Jijia Pan, Jun Xu, Jiebin Dong, Yun Bai, Hongkui Deng, Shicheng Wei. "Injectable Porous Microchips
<1 %

with Oxygen Reservoirs and an Immune-Niche Enhance the Efficacy of CAR T Cell Therapy in Solid Tumors", ACS Applied Materials & Interfaces, 2020
出版物

46 academic.oup.com
网际网络来源 <1%

47 ashpublications.org
网际网络来源 <1%

48 canjhealthtechnol.ca
网际网络来源 <1%

49 jitc.biomedcentral.com
网际网络来源 <1%

50 pure.eur.nl
网际网络来源 <1%

51 Roch Houot, Emmanuel Bachy, Guillaume Cartron, François-Xavier Gros et al. "Axicabtagene ciloleucel in large B cell lymphoma ineligible for autologous stem cell transplantation: the phase 2 ALYCANTE trial", Nature Medicine, 2023
出版物 <1%

52 Sara Ghorashian, Persis Amrolia, Paul Veys. "Open access? Widening access to chimeric antigen receptor (CAR) therapy for ALL", Experimental Hematology, 2018 <1%

出版物

53 Sumel Ashique, Biplab Debnath, Mohhammad Ramzan, Tahreen Taj et al. "A critical review of the synergistic potential of targeting p53 and CAR T cell therapy in cancer treatment", Biomedicine & Pharmacotherapy, 2025 <1 %

出版物

54 Tereza Andreou, Constantina Neophytou, Fotios Mpekris, Triantafyllos Stylianopoulos. "Expanding Immunotherapy Beyond CAR T Cells: Engineering Diverse Immune Cells to Target Solid Tumors", Cancers, 2025 <1 %

出版物

不含引文	开	不含相符结果	关闭
排除参考书目	开		
