# Supplementary material for: CAR‐DC combined with CAR‐T therapy for relapsed/refractory acute myeloid leukaemia: Research progress and future perspectives
Source: Clin Transl Med. 2025 Nov 25;15(12):e70536. doi: 10.1002/ctm2.70536 (PMC12647367; doi:10.1002/ctm2.70536)
Supplement: Supplementary file 3 — Supporting Information [file CTM2-15-e70536-s001.pdf]

CERTIFICATE OF ENGLISH EDITING

This is to certify that the manuscript entitled

CAR-DC Combined with CAR-T Therapy for Relapsed/Refractory Acute Myeloid Leukemia: Research Progress and Future Perspectives

BY:Rui Zhang, Jinlin Zhang, Hongkai Zhang, Mingfeng Zhao

commissioned to us has been carefully edited by a native English-speaking editor at Editeg. The grammar, spelling, and punctuation of the text have carefully been checked and corrected wherever required. We believe that the language of this paper has been considerably improved to meet academic standards. You may please contact us for further queries regarding the editing process.

Date of issue
November 4, 2025

Disclaimer: The changes in the document may be accepted or rejected by the authors at their sole discretion after our editing. Therefore, Editeg would not be responsible for the revisions made to this document after our editing carried on November 4 2025

Editeg Website:https://www.editeg.com

2000+ native English editors:https://www.editeg.com/
