# Supplementary material for: CAR‐DC combined with CAR‐T therapy for relapsed/refractory acute myeloid leukaemia: Research progress and future perspectives
Source: Clin Transl Med. 2025 Nov 25;15(12):e70536. doi: 10.1002/ctm2.70536 (PMC12647367; doi:10.1002/ctm2.70536)
Supplement: Supplementary file 4 — Supporting Information [file CTM2-15-e70536-s003.pdf]

英辑 editeg
SCI论文一站式服务机构

CERTIFICATE OF ENGLISH EDITING

This is to certify that the manuscript entitled

CAR-DC Combined with CAR-T Therapy for Relapsed/Refractory Acute Myeloid Leukemia: Research Progress and Future Perspectives

BY: Rui Zhang, Jinlin Zhang, Hongkai Zhang, Mingfeng Zhao

commissioned to us has been carefully edited by a native English-speaking editor at Editeg. The grammar, spelling, and punctuation of the text have carefully been checked and corrected wherever required. We believe that the language of this paper has been considerably improved to meet academic standards. You may please contact us for further queries regarding the editing process.

LANGUAGE EDITING
EDITEG

Date of issue
October **03, 2025**

Disclaimer: The changes in the document may be accepted or rejected by the authors at their sole discretion after our editing. Therefore, Editeg would not be responsible for the revisions made to this document after our editing carried on October **03 2025**

Editeg Website:https://www.editeg.com

2000+ native English editors:https://www.editeg.com/
